# Attention-based deep learning network for predicting World Health Organization meningioma grade and Ki-67 expression based on magnetic resonance imaging

**DOI:** 10.1007/s00330-025-11958-7

**Published:** 2025-08-20

**Authors:** Xing Cheng, Huaning Li, Chen Li, Jintan Li, Zijie Liu, Xiao Fan, Chenfei Lu, Kefan Song, Zhiyan Shen, Zhichao Wang, Qing Yang, Junxia Zhang, Jianxing Yin, Chunfa Qian, Yongping You, Xiefeng Wang

**Affiliations:** 1 Department of Neurosurgery, The First Affiliated Hospital of Nanjing Medical University, Nanjing, China; 2 Department of Neurosurgery, The Affiliated Brain Hospital of Nanjing Medical University, Nanjing, China; 3 The First Clinical School of Nanjing Medical University, Nanjing, China; 4 Institute for Brain Tumors, Jiangsu Collaborative Innovation Center for Cancer Personalized Medicine, Nanjing Medical University, Nanjing, China

**Keywords:** Meningioma, Ki-67 expression, Deep learning, Attention mechanisms, Magnetic resonance imaging

## Abstract

**Objectives:**

Preoperative assessment of World Health Organization (WHO) meningioma grading and Ki-67 expression is crucial for treatment strategies. We aimed to develop a fully automated attention-based deep learning network to predict WHO meningioma grading and Ki-67 expression.

**Materials and methods:**

This retrospective study included 952 meningioma patients, divided into training (*n* = 542), internal validation (*n* = 96), and external test sets (*n* = 314). For each task, clinical, radiomics, and deep learning models were compared. We used no-new-Unet (nn-Unet) models to construct the segmentation network, followed by four classification models using ResNet50 or Swin Transformer architectures with 2D or 2.5D input strategies. All deep learning models incorporated attention mechanisms.

**Results:**

Both the segmentation and 2.5D classification models demonstrated robust performance on the external test set. The segmentation network achieved Dice coefficients of 0.98 (0.97–0.99) and 0.87 (0.83–0.91) for brain parenchyma and tumour segmentation. For predicting meningioma grade, the 2.5D ResNet50 achieved the highest area under the curve (AUC) of 0.90 (0.85–0.93), significantly outperforming the clinical (AUC = 0.77 [0.70–0.83], *p* < 0.001) and radiomics models (AUC = 0.80 [0.75–0.85], *p* < 0.001). For Ki-67 expression prediction, the 2.5D Swin Transformer achieved the highest AUC of 0.89 (0.85–0.93), outperforming both the clinical (AUC = 0.76 [0.71–0.81], *p* < 0.001) and radiomics models (AUC = 0.82 [0.77–0.86], *p* = 0.002).

**Conclusion:**

Our automated deep learning network demonstrated superior performance. This novel network could support more precise treatment planning for meningioma patients.

**Key Points:**

***Question*** *Can artificial intelligence accurately assess meningioma WHO grade and Ki-67 expression from preoperative MRI to guide personalised treatment and follow-up strategies*?

***Findings*** *The attention-enhanced nn-Unet segmentation achieved high accuracy, while 2.5D deep learning models with attention mechanisms achieved accurate prediction of grades and Ki-67*.

***Clinical relevance*** *Our fully automated 2.5D deep learning model, enhanced with attention mechanisms, accurately predicts WHO grades and Ki-67 expression levels in meningiomas, offering a robust, objective, and non-invasive solution to support clinical diagnosis and optimise treatment planning*.

**Graphical Abstract:**

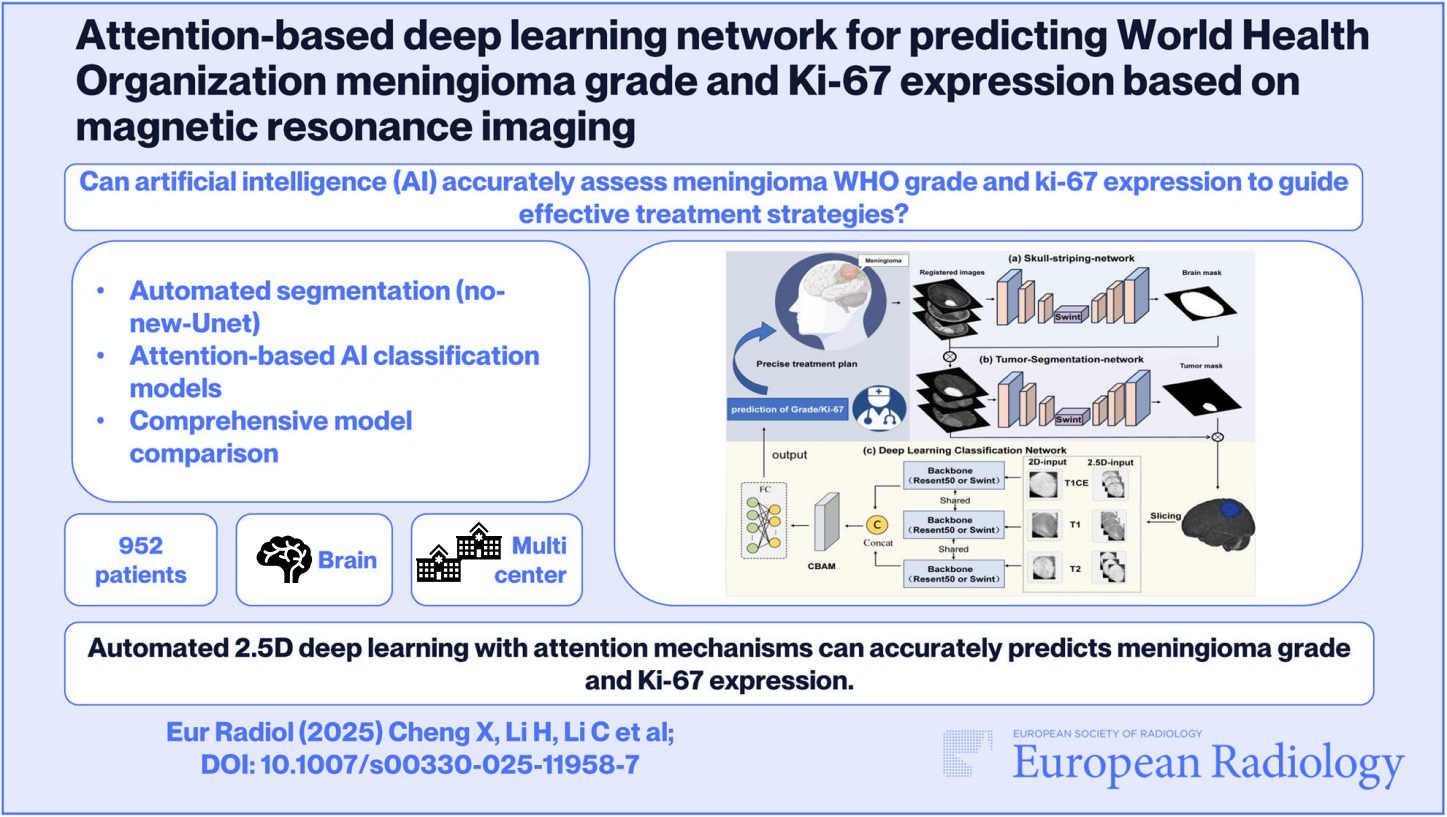

## Introduction

Meningiomas are the most prevalent primary tumour in adults, accounting for 41% of all intracranial neoplasms [1]. The World Health Organization (WHO) classifies meningiomas into three grades: grade I (benign; approximately 80%), grade II (atypical; 15–20%), and grade III (anaplastic; 1–3%). Although most meningiomas are benign, some may exhibit aggressive behaviour. The Ki-67 index, a biomarker of cellular proliferation, serves as a key indicator of tumour aggressiveness [2]. As higher WHO grades and Ki-67 expression levels are associated with greater recurrence rates and poorer prognoses, identifying tumours with high proliferative potential is essential for optimising surgical and follow-up strategies [3–5].

Radiomics transforms high-dimensional imaging data into numerical features, capturing tumour heterogeneity beyond traditional imaging [6]. Previous studies have demonstrated that radiomics can be used preoperatively to predict meningioma grades and Ki-67 expression [7–9], which previously relied on postoperative histopathology. Deep learning, as an advanced machine learning technique, enables the automated extraction of complex features from large datasets and has shown potential for predicting meningioma grades and Ki-67 expression [10–12]. However, most previous studies have been limited to data obtained from a single centre, single-sequence imaging data (T1 or T2), and single-model approaches; moreover, they used manual segmentation and lacked external validation in large samples. Recently, there has been a growing interest in research on novel artificial intelligence technologies in the field of neuroimaging. Attention mechanisms, emerging from recent advancements in deep learning, demonstrate considerable potential. Inspired by the human visual system, they enable models to automatically focus on the most salient regions within input data, potentially improving both the accuracy and efficiency of analysis. These techniques have been applied to brain magnetic resonance imaging (MRI) registration networks, the diagnosis of major depressive disorder, and the prediction of isocitrate dehydrogenase mutation status in gliomas [13–15]. However, their application to the molecular subtyping of meningiomas remains unexplored.

Therefore, we developed a deep learning network that integrates multi-sequence MRI data for automated prediction of WHO meningioma grades and Ki-67 expression. The model incorporated attention mechanisms and automated segmentation to improve tumour characterisation and prediction accuracy. To ensure its generalisability and clinical relevance, we conducted external validation using the largest dataset reported to date.

## Materials and methods

### Study participants

**Fig. 1:**
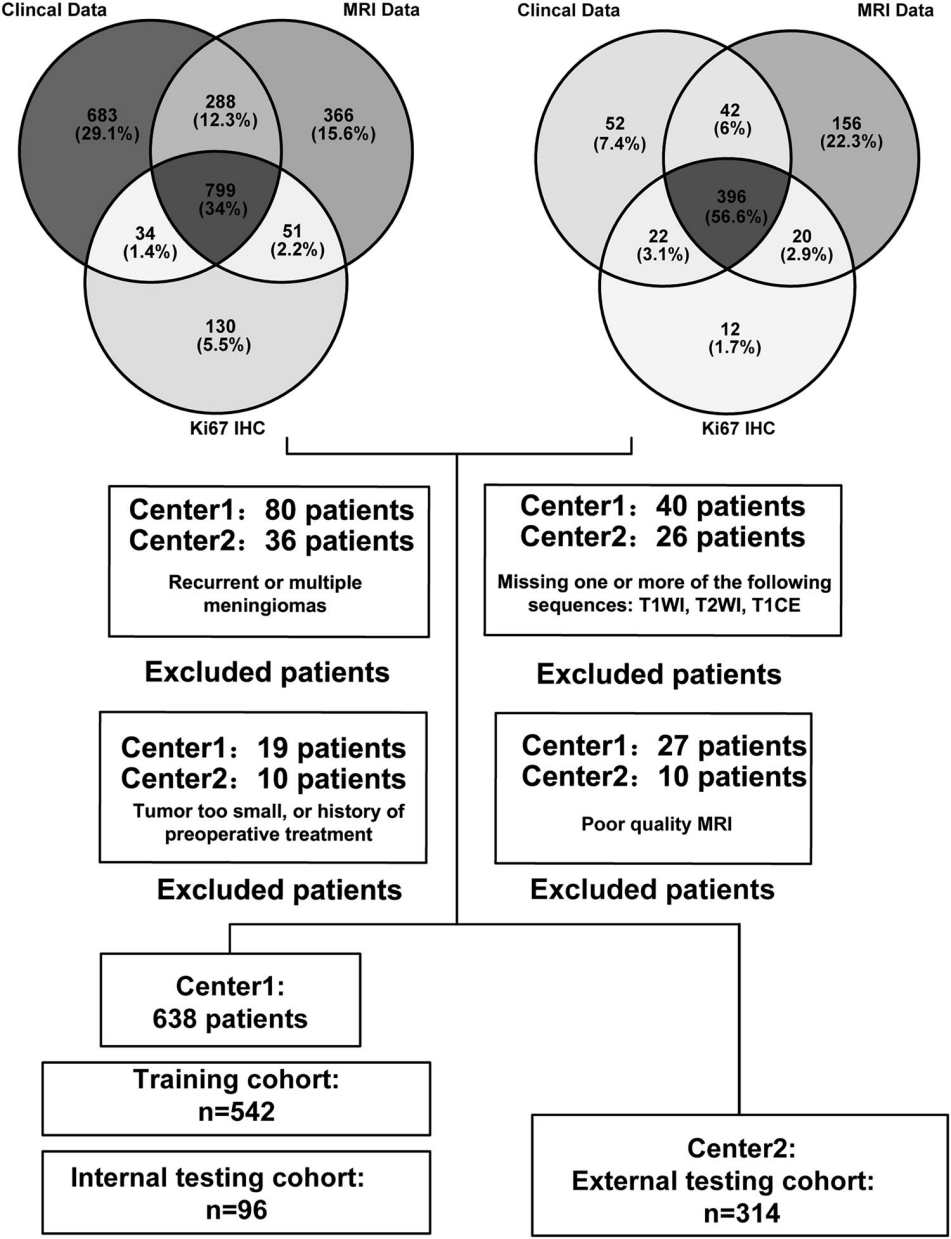
Flowchart of patient selection and data distribution. The Venn diagrams depict the overlap among clinical data, MRI data, and Ki-67 IHC results. The flowchart outlines the exclusion criteria and the number of patients enroled from each centre, culminating in the final training cohort (*n* = 542), internal testing cohort (*n* = 96), and external testing cohorts from Centre 2 (*n* = 314)

We analysed a total of 1195 patients: 799 from Centre 1 (September 2014 to April 2023) and 396 from Centre 2 (February 2018 to April 2023) (Fig. 1). Inclusion criteria were: (1) patients aged over 18 years with a first-time diagnosis of meningioma confirmed via pathology and immunohistochemistry (IHC) following WHO classification; (2) complete preoperative MRI scans, including T1-weighted imaging (T1WI), contrast-enhanced T1-weighted imaging (T1CE), and T2-weighted imaging (T2WI); and (3) no signs of infection on preoperative blood tests. Exclusion criteria were: (1) multiple meningiomas (as their distinct biological characteristics and prognoses could introduce confounding variables); (2) MRI scans exhibiting severe motion artefacts; and (3) history of brain surgery.

Pathological data were obtained from surgically excised tissue samples. According to the 2021 WHO Classification of Tumours of the Central Nervous System, all meningioma samples were re-reviewed and reclassified based on WHO grades. Tumours were categorised into two groups: WHO grade I tumours were classified as low-grade meningiomas, while WHO grade II and III tumours were collectively classified as high-grade meningiomas. The Ki-67 labelling index for all patients was assessed using immunohistochemical (IHC) staining and independently reviewed by a neuropathologist with 16 years of experience. Previous studies have shown that meningioma patients with a Ki-67 labelling index of 5% or higher exhibit higher proliferative potential and an increased risk of recurrence. Therefore, we divided the samples into two groups: high expression (Ki-67 ≥ 5%) and low expression (Ki-67 < 5%) [4, 5, 16, 17].

The study was approved by the ethics committee of two hospitals. Written informed consent was waived because of the retrospective nature of the study.

### MRI data acquisition and preprocessing

Preoperative imaging was conducted for all patients using 3.0 T MRI scanners. Detailed scanning parameters are provided in Supplementary Tables 1–4.

The image preprocessing pipeline included the following steps: (1) advanced normalisation tools were used to rigidly register the T1CE sequence images by adjusting spatial positioning and resampling the images to Montreal Neurological Institute (MNI) space (https://nist.mni.mcgill.ca/atlases/) with the dimensions of 193 × 229 × 193 and a voxel size of 1 × 1 × 1 mm. The T1WI and T2WI scans were rigidly registered to the T1CE image space to ensure alignment within each patient’s image set. (2) Skull stripping was conducted using our trained no-new-Unet (nnU-Net) model on the T1CE images, which were then non-linearly registered to MNI152 standard space. The resulting transformation matrix was subsequently applied to the T1WI and T2WI images to ensure spatial standardisation across all images [18, 19]. (3) N4 bias field correction was applied to all images to correct for low-frequency intensity non-uniformity. (4) For signal intensity standardisation, we compared *Z*-score standardisation and WhiteStripe normalisation [20]. WhiteStripe normalisation identifies normal-appearing white matter, calculates its intensity mean and standard deviation, and then linearly transforms the entire image using these parameters. Our results indicated that the WhiteStripe method was most effective in improving image comparability across the different centres, subjects, and scanning protocols (as illustrated in Supplementary Fig. 1). Furthermore, this study rigorously adheres to the Checklist for Artificial Intelligence in Medical Imaging (CLAIM) guidelines and has comprehensively evaluated the radiomics quality score assessment (Supplementary Methods 5).

### Construction of the clinical and radiomics models

For each task (WHO grade prediction and Ki-67 expression prediction), we developed separate clinical and radiomics models. The clinical model incorporated demographic characteristics (e.g., age and sex), imaging features (e.g., oedema, dural tail sign, mushroom sign, and adjacent venous sinus invasion), and preoperative haematological parameters. A comprehensive list of factors is provided in Table 1. Missing haematological data were imputed using multiple imputation (Supplementary Fig. 2).

**Table 1:** Training and test cohort datasets

Feature	Centre 1			Centre 2	*p* value
	Total	Training set	Testing set (internal)	External testing set	
Age (years)					
Mean (SD)	56.1 (11.3)	56.4 (11.3)	54.3 (11.4)	58.4 (10.7)	0.319
Median [min, max]	56.0 [18.0, 85.0]	57.0 [18.0, 85.0]	55.5 [31.0, 75.0]	58.0 [18.0, 87.0]	0.319
Sex					0.660
Female	433 (67.9%)	364 (67.2%)	69 (71.9%)	234 (74.5%)	
Male	205 (32.1%)	178 (32.8%)	27 (28.1%)	80 (25.5%)	
WHO					0.818
WHO I	384 (60.2%)	326 (60.1%)	58 (60.4%)	251 (79.9%)	
WHO II and III	254 (39.8%)	216 (39.8%)	38 (39.6%)	63 (20.0%)	
Laterality					0.915
Left	294 (46.1%)	246 (45.4%)	48 (50.0%)	144 (45.9%)	
Middle	69 (10.8%)	58 (10.7%)	11 (11.5%)	36 (11.5%)	
Right	275 (43.1%)	238 (43.9%)	37 (38.5%)	134 (42.7%)	
Region					0.958
Cranial base	174 (27.3%)	149 (27.5%)	25 (26.0%)	123 (39.2%)	
Non-cranial base	464 (72.7%)	393 (72.5%)	71 (74.0%)	191 (60.8%)	
Max diameter					0.895
< 4 cm	353 (55.3%)	302 (55.7%)	51 (53.1%)	191 (60.8%)	
≥ 4 cm	285 (44.7%)	240 (44.3%)	45 (46.9%)	123 (39.2%)	
Oedema					0.348
None	237 (37.1%)	195 (36.0%)	42 (43.8%)	156 (49.7%)	
Yes	401 (62.9%)	347 (64.0%)	54 (56.2%)	158 (50.3%)	
Bone invasion					0.337
None	571 (89.5%)	481 (88.7%)	90 (93.8%)	265 (84.4%)	
Yes	67 (10.5%)	61 (11.3%)	6 (6.2%)	49 (15.6%)	
Meningeal tail sign					0.792
None	467 (73.2%)	394 (72.7%)	73 (76.0%)	245 (78.0%)	
Yes	171 (26.8%)	148 (27.3%)	23 (24.0%)	69 (22.0%)	
Mushroom sign					0.991
None	613 (96.1%)	521 (96.1%)	92 (95.8%)	300 (95.5%)	
Yes	25 (3.9%)	21 (3.9%)	4 (4.2%)	14 (4.5%)	
Venous sinus invasion					0.943
None	552 (86.5%)	470 (86.7%)	82 (85.4%)	262 (83.4%)	
Yes	86 (13.5%)	72 (13.3%)	14 (14.6%)	52 (16.6%)	
Ki67					0.998
< 5%	241 (37.8%)	205 (37.8%)	36 (37.5%)	168 (53.5%)	
≥ 5%	397 (62.2%)	337 (62.2%)	60 (62.5%)	146 (46.5%)	

*p* values were calculated to compare the overall dataset, training set, and internal testing set. Chi-square tests or *t*-tests were used, as appropriate

*SD* standard deviation

For the radiomics models, features were extracted exclusively from the segmented tumour regions obtained from our automated segmentation network. We extracted 3157 radiomics features from the three MRI sequences using the Pyradiomics Module (https://github.com/Radiomics/pyradiomics; Supplementary Fig. 3). This approach ensured consistency and reproducibility in feature extraction while avoiding the variability potentially introduced by manual segmentation.

All four models utilised the same machine learning framework but with task-specific feature selection and optimisation. We used a support vector machine because it has previously been shown to outperform other models [21–24]. Detailed feature selection methods, parameter configurations, and training strategies are provided in the Supplementary Methods Section 3 and key radiomics features are further detailed in Supplementary Tables 5 and 6.

### Development of the deep learning segmentation network

Accurate delineation of regions of interest and skull stripping are crucial but labour-intensive tasks in brain tumour imaging analysis. To ensure reliability, brain masks were initially generated using a high-definition brain extraction tool for skull stripping [25]. Two neuroradiologists (10 years and 15 years of experience) independently annotated datasets from Centres 1 and 2 using three-dimensional (3D) Slicer 5.0.3 (https://www.slicer.org/). Annotations involved refining brain masks and delineating tumour regions. A third expert with 20 years of experience resolved discrepancies, ensuring consistency and accuracy.

**Fig. 2:**
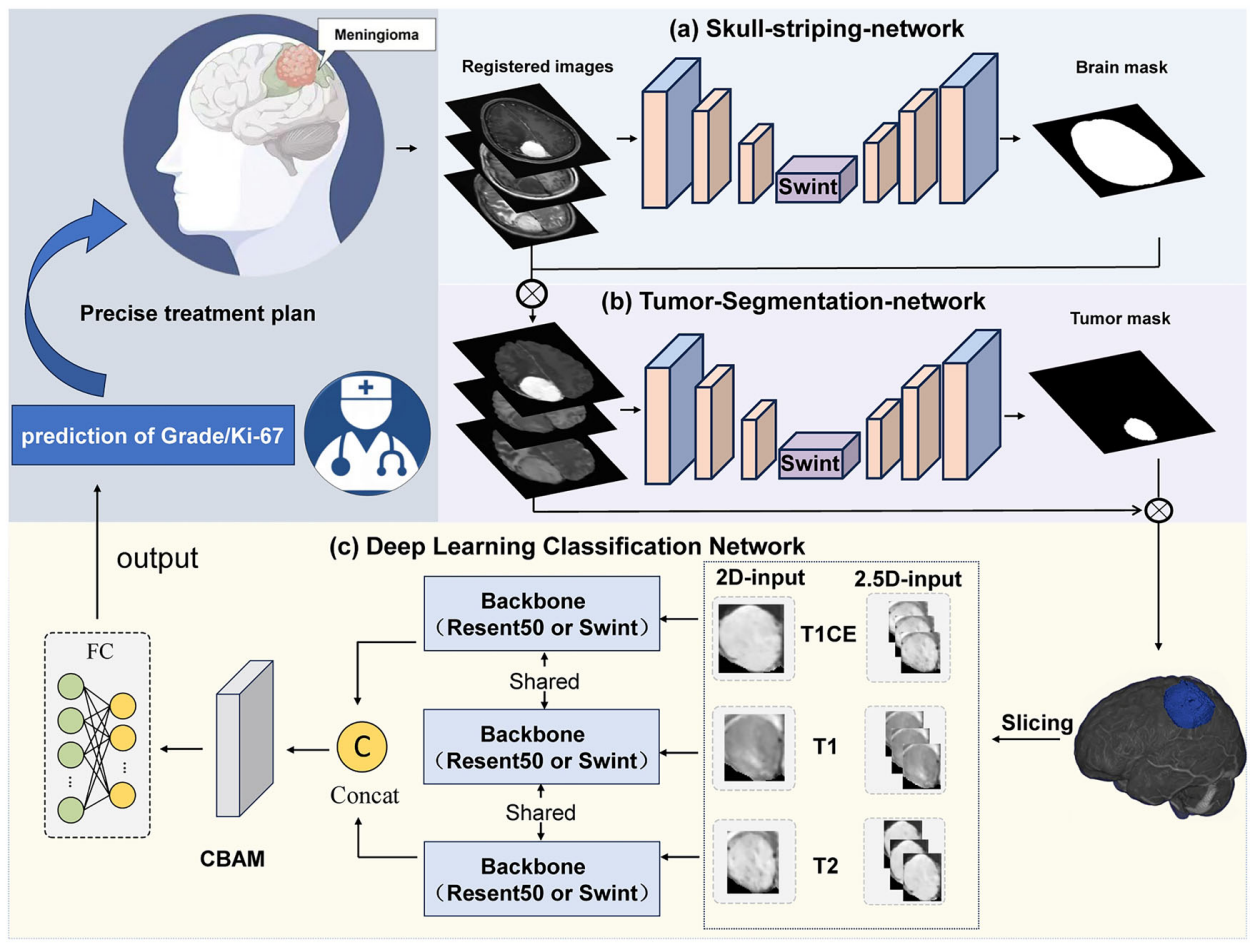
Workflow of the fully automated deep learning network. **a** Skull-stripping network: registered MRI images (T1, T1CE, and T2 sequences) are fed into an nnU-Net model enhanced with attention mechanisms to generate a brain parenchyma segmentation mask, which is used for skull stripping. **b** Tumour segmentation network: After skull stripping, the segmented brain image is processed by another nnU-Net model to generate a tumour segmentation mask, which serves as an input reference for the classification network. **c** Deep learning classification network: based on the segmentation results, 2D slices are extracted from the largest tumour mask and input into either the ResNet50 or Swin Transformer backbone. Features from the three sequences are extracted, concatenated, and enhanced by CBAM, leading to the final classification prediction

We developed automated segmentation models using the nnU-Net framework optimised for dataset-specific characteristics [19]. Data from Centre 1, including MRI T1WI, T1CE, T2WI images, brain masks, and tumour annotations, were used to train two nnU-Net models: one for brain parenchyma segmentation and another for tumour region segmentation (Fig. 2a, b). Both models used a 3D full-resolution U-Net architecture, incorporating Swin Transformer modules in the low-resolution pathway to enhance segmentation accuracy by capturing global semantic information (Supplementary Fig. 4). Five-fold cross-validation was performed over 500 epochs. The trained models were tested on data from Centre 2 to evaluate their generalisation capability. Learning curves for the models are provided in Supplementary Figs. 5 and 6.

### Development of the deep learning classification network

Consistent with the clinical and radiomics approaches, dedicated deep learning models were trained for each of the two prediction tasks: WHO grade classification and Ki-67 expression prediction. Two backbone networks were used for classification: ResNet50, based on convolutional neural networks, and Swin Transformer, built on attention mechanisms. The architecture was modified to concatenate features from all three MRI sequence images, incorporating a convolutional block attention module (CBAM) for both channel and spatial attention. Final classifications were generated by a fully connected output layer.

Based on the segmentation results, input data for the classification models were prepared using two methods: 2D and 2.5D, both utilising axial slices. The 2D model used tumour bounding boxes from the largest slice (slice *n*) across all MRI sequence images, whereas the 2.5D model included adjacent slices (*n* + 2 and *n* − 2) to capture both local tumour features and the surrounding context (Fig. 2c). Preprocessing steps comprised resizing, normalisation, and tensor conversion.

To mitigate data imbalance, we used PyTorch’s ImbalancedDatasetSampler to achieve balanced class representation in each batch. Data augmentation techniques, including random flipping, rotation, and Gaussian noise addition, were applied during training to enhance data diversity and reduce overfitting, whereas only basic preprocessing was used for validation and testing to simulate real-world conditions. Learning curves for the 2D and 2.5D models are provided in Supplementary Figs. 7 and 8, alongside detailed training configurations, which are provided in the Supplementary Methods Section 4.

To enhance interpretability, gradient-weighted class activation mapping (Grad-CAM) was used to generate heatmaps highlighting regions that influenced the model’s predictions. Two representative cases were selected: Case 1, a low-grade tumour with low Ki-67 expression; Case 2, a high-grade tumour with high Ki-67 expression. These cases encompassed different levels of malignancy and proliferative potential, thus providing a comprehensive evaluation of the models.

### Statistical analysis

MRI processing and statistical analyses were primarily conducted using Python (v3.9) (https://www.python.org) and R (v4.3.2; http://www.Rproject.org). For comparisons of categorical variables, we used chi-square or Fisher’s exact tests, and for comparisons of continuous variables, we used Mann–Whitney *U*-tests and independent *t*-tests. The performance of the predictive models was evaluated using the area under the receiver operating characteristic (ROC) curve (AUC), accuracy, sensitivity, specificity, and F1 score. Comparisons between AUCs were conducted using DeLong’s test. A *p* < 0.05 was considered statistically significant.

## Results

### Patient characteristics

We recruited a total of 1195 patients in the study. Of these, 638 patients from Centre 1 and 314 from Centre 2 fulfilled the eligibility criteria and were included in the final analysis (Fig. 1). In Centre 1, 542 patients were allocated to the training set and 96 to the internal testing set (mean age 56.1 years, 67.9% women). The external testing set from Centre 2 consisted of 314 patients (mean age 58.4 years, 74.5% women). Age and sex distributions were comparable between the two centres. The baseline characteristics of the patients are summarised in Table 1.

### Performance of the deep learning segmentation network

The nnU-Net model integrated with Swin Transformer demonstrated excellent performance on the validation set from Centre 1, with mean Dice coefficients of 0.98 ± 0.01 for brain parenchyma and 0.92 ± 0.03 for tumour segmentation. To assess generalisability, we tested the model on an independent dataset from Centre 2. The brain parenchyma segmentation achieved a Dice coefficient of 0.98 (95% confidence interval (CI): 0.97–0.99) and a volumetric similarity of 0.99 (95% CI: 0.98–1.00). These metrics for tumour segmentation were 0.87 (95% CI: 0.83–0.91) and 0.92 (95% CI: 0.89–0.95), respectively.

### Comparative analysis of the grade and Ki-67 proliferation index prediction models

The performance metrics for each model are presented in Table 2. The traditional models, including the clinical and radiomics approaches, demonstrated moderate predictive accuracy across both tasks. For grade prediction, the clinical model achieved an accuracy of 69% (95% CI: 59–78%) and an AUC of 0.72 (95% CI: 0.61–0.83) on the internal test set and an accuracy of 76% (95% CI: 71–81%) with an AUC of 0.77 (95% CI: 0.70–0.83) on the external test set. The radiomics model showed slightly higher performance, with an accuracy of 77% (95% CI: 72–82%) and an AUC of 0.80 (95% CI: 0.75–0.85) on the external test set. Similarly, for predicting the Ki-67 index, the clinical model exhibited relatively poor performance, with an accuracy of 59% (95% CI: 54–65%) and an AUC of 0.76 (95% CI: 0.71–0.81), whereas the radiomics model achieved 74% (95% CI: 69–78%) accuracy and an AUC of 0.82 (95% CI: 0.77–0.86). Despite some improvement in performance using radiomics features, the traditional models were unable to adequately capture the complexity of imaging features related to tumour grading and proliferation.

**Table 2:** Performance comparison of the different models in predicting the WHO grade and Ki-67 index

Model and stage (WHO grade)		Accuracy	Sensitivity	Specificity	AUC	*p* value1	*p* value2
Val (CV) (*n* = 542)	2.5D ResNet50	0.77 [0.73, 0.80]	0.84 [0.78, 0.89]	0.72 [0.67, 0.77]	0.84 [0.81, 0.87]	reference	0.008*
	2.5D Swin-T	0.75 [0.72, 0.79]	0.85 [0.80, 0.89]	0.69 [0.64, 0.74]	0.80 [0.76, 0.84]	0.008*	Reference
	2D ResNet50	0.69 [0.66, 0.73]	0.82 [0.77, 0.87]	0.61 [0.55, 0.66]	0.76 [0.71, 0.80]	< 0.001*	0.014*
	2D Swin-T	0.76 [0.72, 0.80]	0.77 [0.71, 0.83]	0.75 [0.70, 0.80]	0.80 [0.76, 0.84]	0.012*	0.866
	Clinical	0.66 [0.62, 0.70]	0.32 [0.26, 0.38]	0.89 [0.85, 0.92]	0.71 [0.66, 0.75]	< 0.001*	< 0.001*
	Radiomics	0.73 [0.69, 0.77]	0.55 [0.48, 0.61]	0.86 [0.82, 0.89]	0.82 [0.78, 0.85]	0.195	0.450
Internal testing set (*n* = 96)	2.5D ResNet50	0.76 [0.68, 0.84]	0.61 [0.44, 0.76]	0.86 [0.77, 0.94]	0.86 [0.78, 0.93]	Reference	0.086
	2.5D Swin-T	0.72 [0.62, 0.80]	0.79 [0.65, 0.91]	0.67 [0.55, 0.79]	0.80 [0.71, 0.88]	0.086	Reference
	2D ResNet50	0.67 [0.57, 0.77]	0.84 [0.71, 0.95]	0.55 [0.42, 0.69]	0.77 [0.67, 0.87]	0.023*	0.405
	2D Swin-T	0.71 [0.61, 0.79]	0.63 [0.48, 0.79]	0.76 [0.64, 0.87]	0.75 [0.64, 0.85]	0.002*	0.106
	Clinical	0.69 [0.59, 0.78]	0.34 [0.19, 0.49]	0.91 [0.84, 0.98]	0.72 [0.61, 0.83]	0.029*	0.211
	Radiomics	0.65 [0.55, 0.74]	0.45 [0.29, 0.60]	0.78 [0.67, 0.87]	0.74 [0.65, 0.84]	0.014*	0.134
External testing set (*n* = 314)	2.5D ResNet50	0.88 [0.84, 0.91]	0.57 [0.44, 0.69]	0.96 [0.93, 0.98]	0.90 [0.85, 0.93]	Reference	0.922
	2.5D Swin-T	0.84 [0.80, 0.88]	0.86 [0.77, 0.94]	0.84 [0.79, 0.88]	0.90 [0.84, 0.94]	0.922	Reference
	2D ResNet50	0.70 [0.65, 0.75]	0.87 [0.79, 0.95]	0.65 [0.60, 0.71]	0.84 [0.78, 0.89]	0.030*	0.031*
	2D Swin-T	0.76 [0.71, 0.81]	0.70 [0.58, 0.80]	0.77 [0.72, 0.82]	0.84 [0.79, 0.88]	0.010*	0.007*
	Clinical	0.76 [0.71, 0.81]	0.56 [0.44, 0.67]	0.81 [0.76, 0.85]	0.77 [0.70, 0.83]	< 0.001*	< 0.001*
	Radiomics	0.77 [0.72, 0.82]	0.40 [0.27, 0.53]	0.86 [0.81, 0.90]	0.80 [0.75, 0.85]	< 0.001*	0.001*
**Model and stage (Ki67)**		**Accuracy**	**Sensitivity**	**Specificity**	**AUC**	***p*** **value1**	***p*** **value2**
Val (CV) (*n* = 542)	2.5D ResNet50	0.80 [0.76, 0.83]	0.85 [0.82, 0.89]	0.71 [0.64, 0.77]	0.83 [0.79, 0.87]	Reference	0.210
	2.5D Swin-T	0.82 [0.79, 0.85]	0.93 [0.90, 0.96]	0.64 [0.58, 0.70]	0.85 [0.82, 0.88]	0.210	Reference
	2D ResNet50	0.79 [0.75, 0.82]	0.93 [0.90, 0.95]	0.56 [0.49, 0.63]	0.84 [0.80, 0.87]	0.539	0.546
	2D Swin-T	0.80 [0.77, 0.84]	0.92 [0.90, 0.95]	0.60 [0.54, 0.67]	0.84 [0.80, 0.87]	0.647	0.381
	Clinical	0.64 [0.60, 0.68]	0.88 [0.84, 0.91]	0.25 [0.20, 0.31]	0.68 [0.64, 0.73]	< 0.001*	< 0.001*
	Radiomics	0.80 [0.76, 0.83]	0.87 [0.83, 0.90]	0.68 [0.61, 0.74]	0.87 [0.83, 0.90]	0.028*	0.291
Internal testing set (*n* = 96)	2.5D ResNet50	0.81 [0.74, 0.89]	0.90 [0.82, 0.97]	0.67 [0.51, 0.81]	0.86 [0.78, 0.93]	Reference	0.458
	2.5D Swin-T	0.75 [0.66, 0.83]	0.90 [0.82, 0.97]	0.50 [0.32, 0.67]	0.84 [0.76, 0.91]	0.458	Reference
	2D ResNet50	0.71 [0.62, 0.80]	0.90 [0.82, 0.97]	0.39 [0.24, 0.55]	0.81 [0.73, 0.90]	0.201	0.481
	2D Swin-T	0.75 [0.66, 0.83]	0.90 [0.82, 0.97]	0.50 [0.34, 0.66]	0.82 [0.74, 0.90]	0.361	0.642
	Clinical	0.69 [0.59, 0.77]	0.90 [0.81, 0.97]	0.33 [0.18, 0.48]	0.74 [0.63, 0.84]	0.020*	0.072
	Radiomics	0.74 [0.66, 0.82]	0.83 [0.73, 0.92]	0.58 [0.42, 0.73]	0.76 [0.66, 0.85]	0.008*	0.040*
External testing set (*n* = 314)	2.5D ResNet50	0.80 [0.75, 0.84]	0.79 [0.72, 0.86]	0.80 [0.74, 0.85]	0.87 [0.83, 0.91]	Reference	0.113
	2.5D Swin-T	0.81 [0.76, 0.85]	0.90 [0.86, 0.95]	0.72 [0.65, 0.79]	0.89 [0.85, 0.93]	0.113	Reference
	2D ResNet50	0.68 [0.63, 0.74]	0.92 [0.88, 0.96]	0.48 [0.40, 0.56]	0.82 [0.77, 0.87]	0.011*	< 0.001*
	2D Swin-T	0.68 [0.62, 0.72]	0.88 [0.82, 0.93]	0.50 [0.42, 0.57]	0.81 [0.76, 0.85]	0.003*	< 0.001*
	Clinical	0.59 [0.54, 0.65]	0.88 [0.82, 0.93]	0.34 [0.27, 0.41]	0.76 [0.71, 0.81]	< 0.001*	< 0.001*
	Radiomics	0.74 [0.69, 0.78]	0.86 [0.80, 0.91]	0.63 [0.56, 0.70]	0.82 [0.77, 0.86]	0.052	0.002*

*p* values were calculated using the DeLong test. *p* value1 represents the DeLong test *p* values, using 2.5D ResNet50 as the reference model, comparing other models to 2.5D ResNet50. *p* value2 represents the DeLong test *p* values, using 2.5D Swin-Transformer as the reference model, comparing other models to 2.5D Swin-Transformer

*Swin-T* Swin Transformer

* *p* < 0.05

The deep learning models demonstrated superior performance than the traditional models, especially those using the 2.5D approaches. The 2D models showed modest gains over the traditional models on the test sets, with accuracies ranging from 67% to 76% and AUC values ranging from 0.75 to 0.84 for both tasks. However, the 2.5D models achieved even further gains. For grade prediction, the 2.5D ResNet50 achieved an accuracy of 88% (95% CI: 84–91%) and an AUC of 0.90 (95% CI: 0.85–0.93), and the 2.5D Swin Transformer reached 84% (95% CI: 80–88%) accuracy and the same AUC. In predicting the Ki-67 index, the 2.5D Swin Transformer demonstrated the highest accuracy of 81% (95% CI: 76–85%) and an AUC of 0.89 (95% CI: 0.85–0.93).

**Fig. 3:**
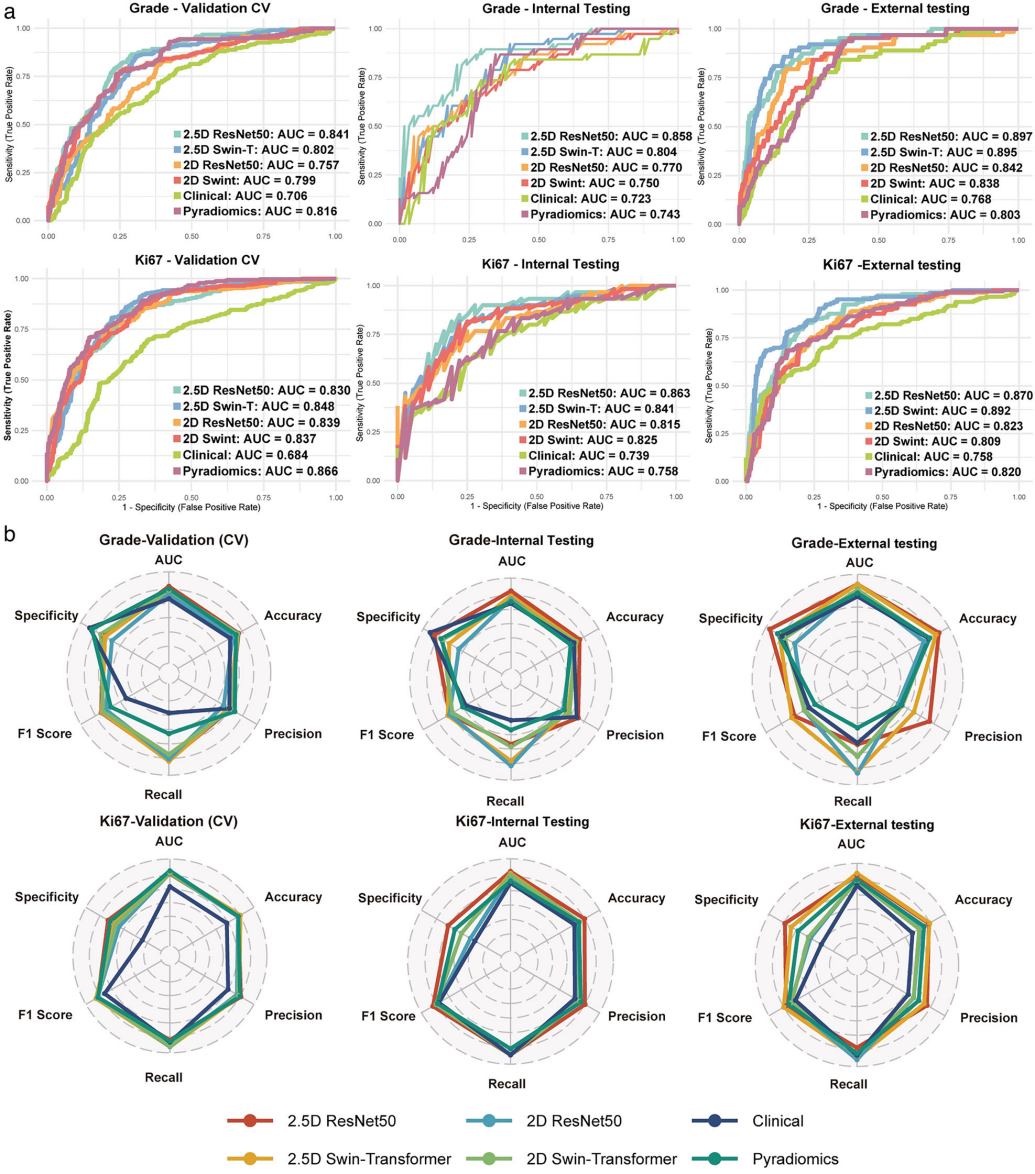
Prediction performance for meningioma grade and Ki-67 expression. **a** The ROC curves of the 2.5D ResNet50 model, 2.5D Swin Transformer model, 2D ResNet50 model, 2D Swin Transformer model, clinical model, and Pyradiomics Model in the cross-validation set, internal testing set, and external testing set. **b** Radar charts comparing the six models across various metrics (AUC, accuracy, specificity, precision, F1 score, and recall) for the same tasks and datasets

Figure 3a illustrates the above results and shows that the 2.5D models consistently achieved higher AUC values across all datasets for both tasks. Figure 3b further demonstrates the superior predictive capability of the 2.5D models, as reflected in metrics, such as accuracy, recall, and F1 score. Additional performance evaluation plots, such as decision curve analysis curves (DCA), calibration curves, and other relevant metrics, are provided in Supplementary Figs. 9–12.

To evaluate the fairness of our models across different demographic groups, particularly addressing the sample imbalance characterised by a higher proportion of female patients with meningioma, we conducted stratified analyses based on gender and age. In the male subgroup of the external test set (*n* = 80), our 2.5D models demonstrated excellent performance in both tasks: the 2.5D ResNet50 achieved an area under the curve (AUC) of 0.94 (95% CI: 0.87–0.98) and an accuracy of 89% (95% CI: 81–95%) for WHO grading prediction. Similarly, within this subgroup, the 2.5D Swin-T achieved an AUC of 0.91 (95% CI: 0.82–0.98) and an accuracy of 89% (95% CI: 81–95%) for the Ki-67 prediction task. Analyses across various age subgroups further revealed that both 2.5D models maintained consistent and robust predictive performance. These findings demonstrate that, notwithstanding the gender imbalance, our 2.5D models provide fair and high-accuracy predictions across diverse demographic groups. Detailed stratified analyses are presented in Supplementary Tables 7–10.

### Interpretability of clinical, radiomics, and deep learning models

The Shapley additive explanation analysis offered valuable insight into the decision-making processes of the clinical and radiomics models (Supplementary Fig. 13). Predictions from the clinical model were primarily driven by imaging features, such as tumour diameter and oedema, as well as preoperative haematological parameters, such as chloride and haemoglobin levels, highlighting the significance of integrating multimodal data. The radiomics models were dependent on advanced texture features, such as the Grey Level Dependence Matrix and Grey Level Size Zone Matrix, demonstrating the potential for capturing tumour microstructure. Nevertheless, these traditional models had limitations in explaining complex biological behaviours.

**Fig. 4:**
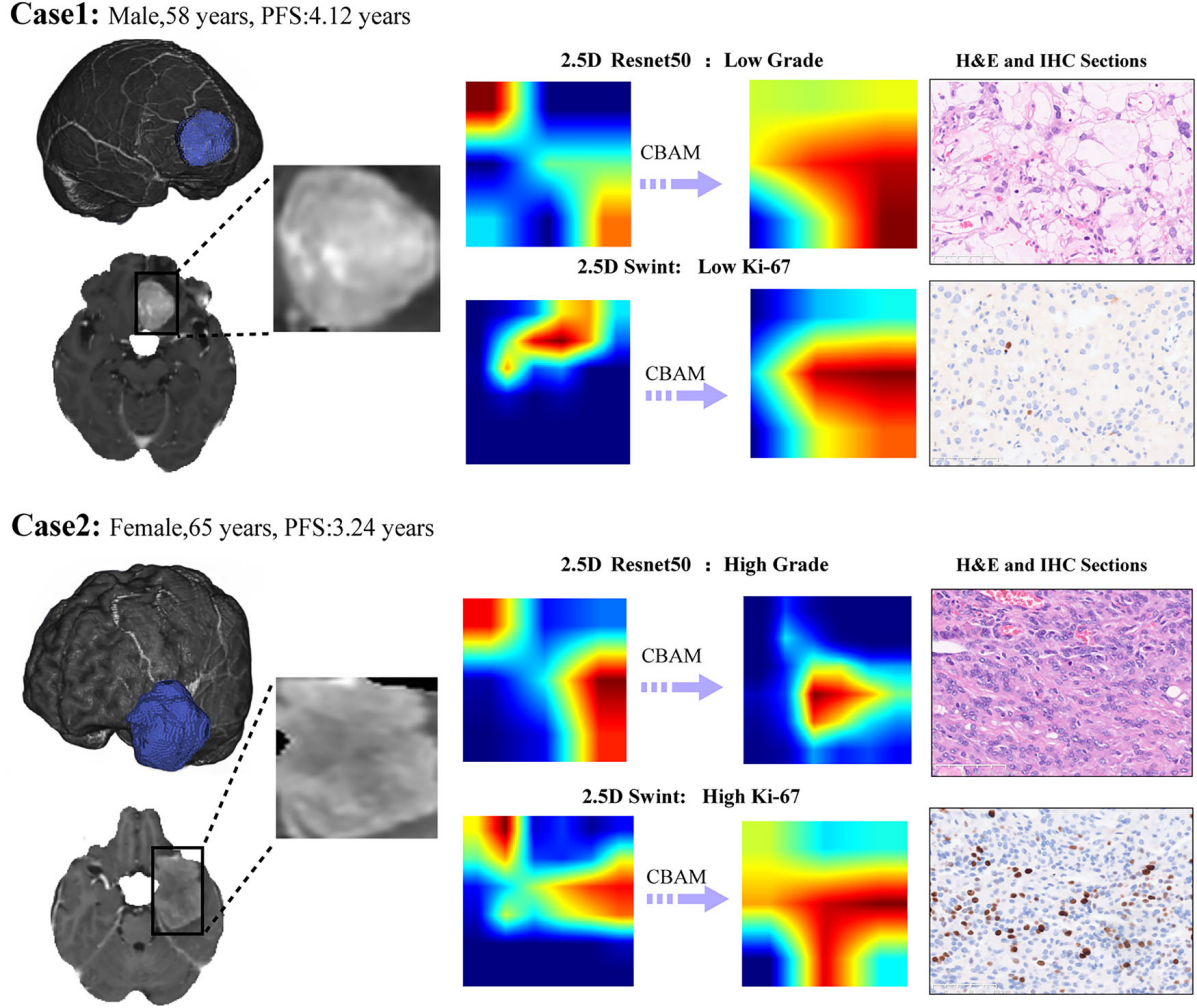
Grad-CAM visualisation for the 2.5D ResNet50 and 2.5D Swin Transformer models. The Grad-CAM heatmaps illustrate how the CBAM refocused the models on key internal tumour regions. In Case 1, the model primarily focused on the uniform areas of the tumour, indicating lower grade and lower Ki-67 expression, accompanied by longer PFS. In Case 2, the model shifted its focus to more heterogeneous regions, suggesting higher aggressiveness, with predictions indicating a higher grade and elevated Ki-67 expression, aligning with clinical observations of shorter PFS. The model’s predictions were consistent with the findings from haematoxylin and eosin staining and immunohistochemical analysis

The 2.5D deep learning models showed superior interpretability, especially when incorporating the CBAM, which enabled a greater focus on critical tumour regions. Notably, the introduction of CBAM redirected the models’ attention to the internal regions of the tumours (Fig. 4). For instance, for Case 1, the model focused primarily on the tumour itself, which was characterised by uniform enhancement and a regular shape, indicating a higher likelihood of benignity and prolonged progression-free survival (PFS). In contrast, in Case 2, the CBAM-enhanced model focused more on the heterogeneous internal regions of the tumour, which exhibited irregular enhancement. This shift in focus aligns with the potential for invasive tumour behaviour and corresponds to the shorter PFS of patients with high-grade tumours with elevated Ki-67 expression.

## Discussion

We developed an innovative, fully automated attention-based 2.5D deep learning model that incorporated attention mechanisms to predict WHO grades and Ki-67 expression levels of meningiomas. This is the first study to implement an attention mechanism-based model on a large-scale dual-centre dataset, consisting of 952 cases. This approach addresses the limitations of existing models, particularly regarding automation, interpretability, and generalisability.

To address imaging variations due to different sources and parameters, we compared *Z*-score and White Stripe normalisation methods. The White Stripe method yielded more consistent signal intensity distributions, which ensured stability across multi-centre and multi-device settings. This comparison offered important insight into optimising image normalisation techniques for neuroimaging workflows.

Clinical indicators, such as imaging and haematological characteristics, have shown promise in predicting WHO meningioma grades. A single-centre study by Zhao et al achieved an AUC of 0.726 using preoperative haematological characteristics, which is similar to the AUC of 0.768 achieved by our clinical model [7]. This suggests that clinical indicators alone may not fully capture the complexity of tumour biology, highlighting the need for advanced predictive methods.

Radiomics extracts high-dimensional texture features from medical images to enable non-invasive and objective tumour characterisation. Several studies to date have demonstrated the effectiveness of using radiomics to predict WHO grades and Ki-67 expression of meningiomas. In a single-centre study by Khanna et al, the AUC for predicting Ki-67 expression in the test set was 0.83 [16]. In another single-centre study, Zhao et al achieved an AUC of 0.860 for predicting WHO grade [7]. However, these approaches relied on manual segmentation and carried a risk of overfitting due to limited external validation, as well as significant inter-model feature variability. In our study, we achieved AUCs of 0.74 and 0.76 for WHO grade and Ki-67 prediction, respectively, in the internal test set. These findings underscore the need to enhance predictive accuracy and model generalisability and the value of advanced techniques to overcome the limitations of traditional radiomics.

Deep learning methods are increasingly being used to predict WHO grades and Ki-67 expression levels of meningiomas. For instance, Chen et al developed a 2D deep learning model using T1CE sequence images and achieved AUCs of 0.669 and 0.591 for predicting WHO grade and Ki-67 expression level, respectively, in an external test cohort of 154 patients [12]. Similarly, Jun et al developed a 3D meningioma grading model using T1CE and T2WI sequence images and achieved an AUC of 0.770 in an external validation set of 61 cases [11]. However, current deep learning approaches often lack comprehensive model comparisons and large-scale external validation, which limits their generalisability. The absence of direct comparisons with traditional methods obscures the performance-complexity trade-off; 2D models may not fully capture the 3D information of MRI data, whereas 3D models face computational and overfitting challenges, especially with limited sample sizes.

To overcome these limitations, we developed a fully automated nnU-Net segmentation model and implemented a 2.5D classification approach. The 2.5D method incorporates information from adjacent slices, which balances the capture of spatial information and computational efficiency. Although 2.5D methods have been shown to be effective in glioma research, this is the first study to apply such methods to meningiomas [26, 27]. We have demonstrated their ability to balance spatial information capture with computational efficiency.

This study pioneers the application of attention mechanisms in predicting meningioma molecular subtypes. We integrated the Swin Transformer into the nnU-Net segmentation network and the CBAM into the classification network. CBAM helps the model determine which feature channels (representing different types of image features) and spatial positions (specific regions in the image) are more important, thereby allocating computational resources to these key areas and effectively focusing on subtle features within the tumour that may correlate with malignancy or proliferative activity. Similarly, the Swin Transformer module in the segmentation network, with its inherent attention mechanism, helps capture long-range dependencies between different image regions, which is crucial for understanding the tumour’s overall structure and its relationship with surrounding tissues. As demonstrated by the Grad-CAM visualisations (Fig. 4), the CBAM enhanced the model’s performance and interpretability by focusing on key tumour regions. These improvements resulted in more accurate predictions of WHO grades and Ki-67 expression.

This study evaluated the calibration performance of the models. While the 2.5D ResNet50 and Swin-Transformer models demonstrated acceptable overall calibration (HL *p* > 0.05) in both internal and external test sets, binned analysis revealed specific local biases, such as the 2.5D ResNet50 model overestimation in the low-risk (0.1–0.2) and underestimation in the moderate-risk (0.5–0.8) intervals in the WHO grading classification task in the external test set, which could lead to unnecessary patient anxiety or influence treatment decisions. Clinicians should be aware of local biases when using these models. Future improvements should focus on incorporating larger sample sizes across diverse populations and implementing calibration techniques such as Platt scaling or isotonic regression to address these specific calibration deficiencies.

DCA curves further validated the clinical utility of our models. For grade prediction, the 2.5D ResNet50 model provided higher net clinical benefit across a wide range of decision thresholds compared to traditional models: at low thresholds (20–30%), it minimised the risk of missing high-grade meningiomas, while at high thresholds (60–70%), it supported more invasive intervention decisions (such as extended resection). Similarly, for Ki-67 prediction, the 2.5D Swin-Transformer effectively identified patients requiring enhanced follow-up at low thresholds (20–30%) and provided a reliable basis for accurate recurrence risk assessment and individualised treatment planning at high thresholds (60–70%). In clinical applications, model predictions should be combined with Grad-CAM visualisations and clinical context to form comprehensive judgments.

Despite the promising results, this study has several limitations. As a retrospective study of data from two centres, selection and information bias may impact the generalisability of the results. Future prospective multi-centre studies with diverse populations and equipment are needed to validate model robustness. Besides, we did not integrate clinical data into the deep learning models due to potential heterogeneity in clinical parameter definitions and measurements across different institutions, which could introduce bias. Future studies with standardised multicentre datasets could explore multimodal models combining clinical and imaging features. Additionally, we only examined two backbone networks (i.e., the ResNet50 and Swin Transformer). Future research should explore a broader range of architectures. Finally, we only focused on conventional MRI sequences, excluding functional imaging techniques, such as diffusion- and perfusion-weighted imaging. Integrating multimodal MRI sequences may further enhance predictive performance and clinical applicability.

The inclusion of a large-scale independent external test set and comparative analysis of multiple models strengthened the reliability of our findings. Our approach can assist clinicians in tumour assessment and treatment planning, which may lead to improvement in patient outcomes. Future work should focus on expanding the research scope, integrating multimodal imaging data, and refining the model to develop more robust diagnostic and treatment tools for meningiomas.

## Supplementary information

Supplementary information

## Abbreviations

AUC: Area under the curve
CBAM: Convolutional block attention module
CI: Confidence interval
Grad-CAM: Gradient-weighted class activation mapping
IHC: Immunohistochemistry
MNI: Montreal Neurological Institute
MRI: Magnetic resonance imaging
nnU-Net: No-new-Unet
PFS: Progression-free survival
ROC: Receiver operating characteristic
T1CE: Contrast-enhanced T1-weighted imaging
T1WI: T1-weighted imaging
T2WI: T2-weighted imaging
WHO: World Health Organization
