# Supplementary material for: Attention-based deep learning network for predicting World Health Organization meningioma grade and Ki-67 expression based on magnetic resonance imaging
Source: Eur Radiol. 2025 Aug 20;36(4):2518–30. doi: 10.1007/s00330-025-11958-7 (PMC13035881; doi:10.1007/s00330-025-11958-7)
Supplement: Supplementary file 1 — Supplementary information [file 330_2025_11958_MOESM1_ESM.pdf]

Attention-Based Deep Learning Network for Predicting World Health Organization Meningioma Grade and Ki-67 Expression Based on Magnetic Resonance Imaging

ELECTRONIC SUPPLEMENTARY MATERIAL

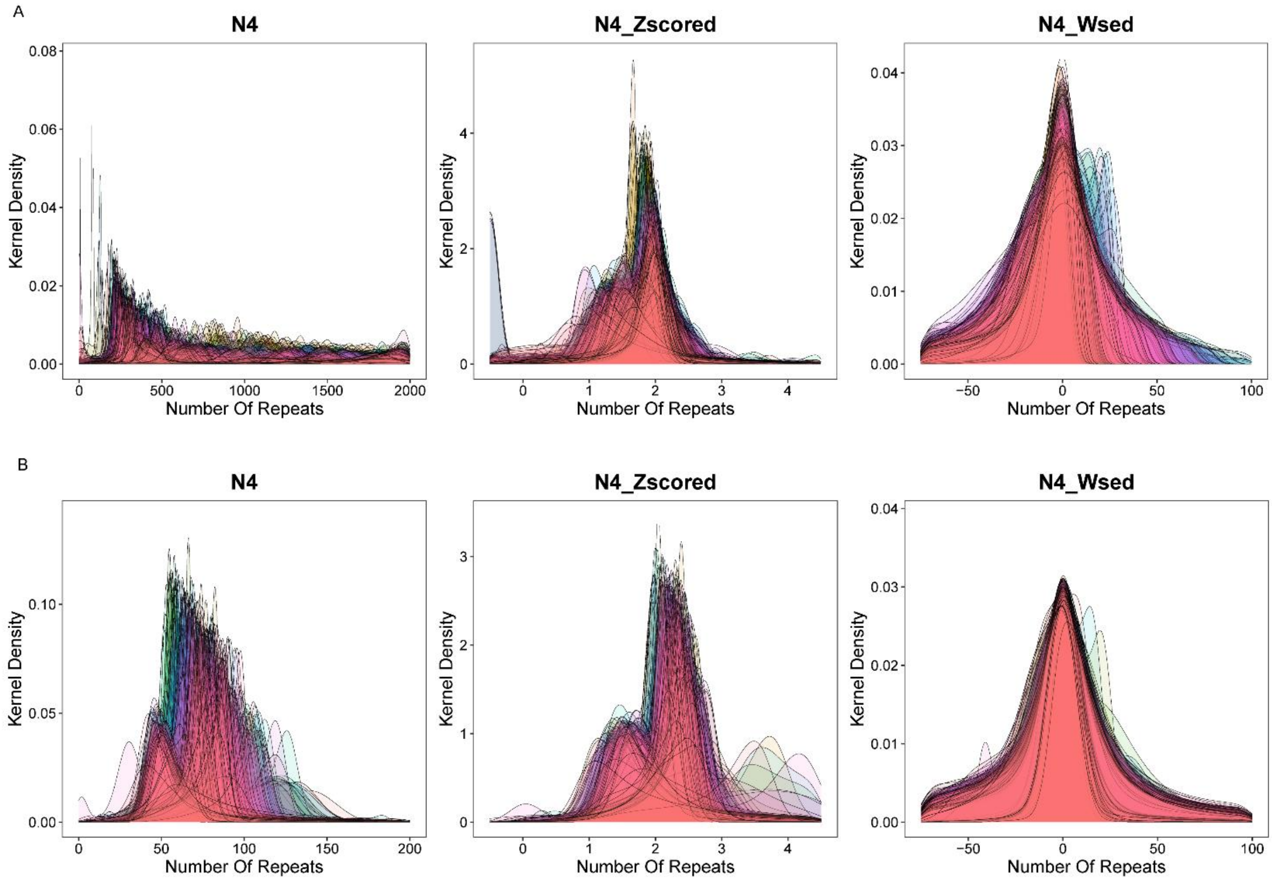

FigureS1: The figures depict the kernel density distributions of signal intensity after N4 correction, N4 correction with Z-score normalization, and N4 correction with WhiteStrip normalization. The WhiteStrip method achieves a more concentrated and consistent signal intensity distribution. Panel A shows data from Center 1, while Panel B shows data from Centre 2.

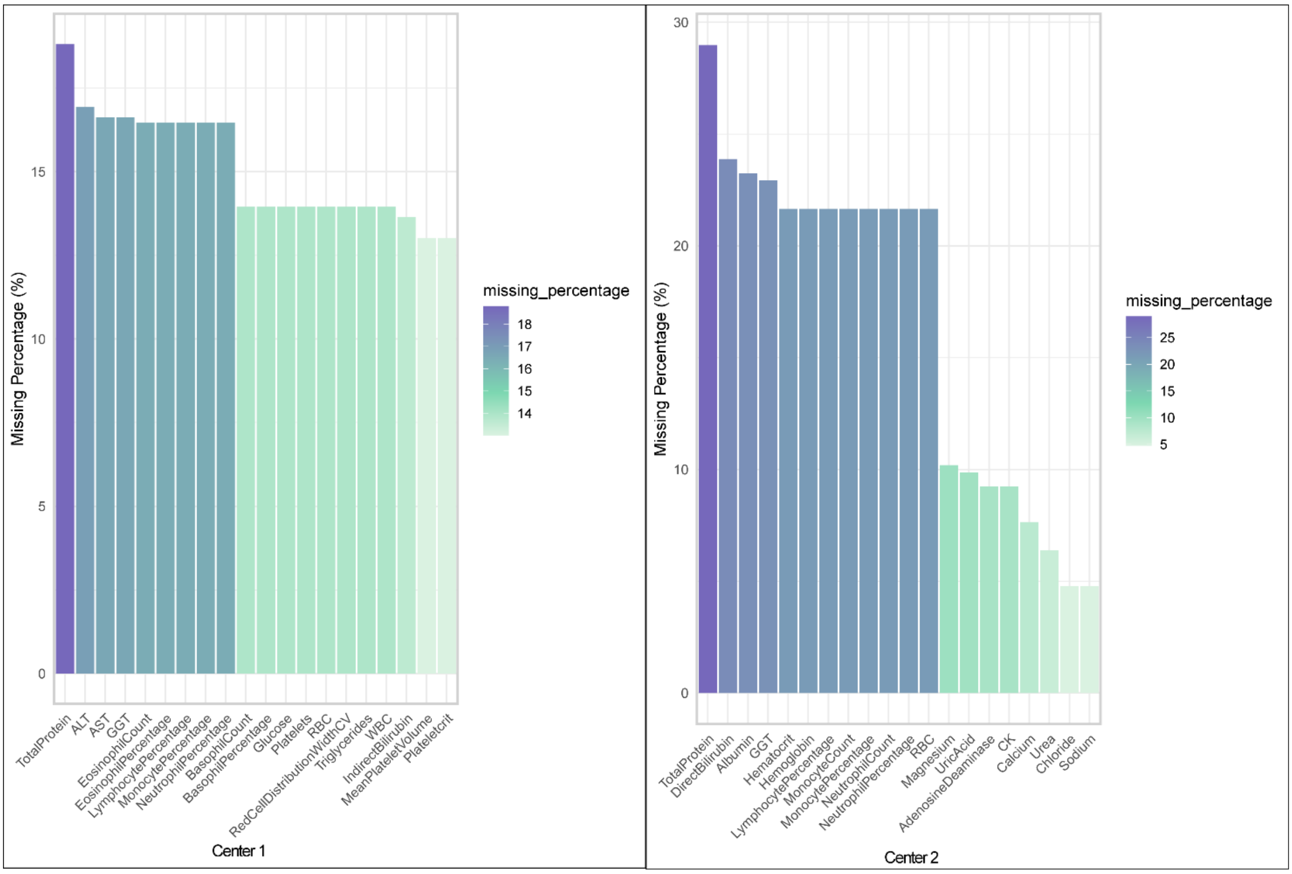

FigureS2: Visualization of missing values in preoperative hematology for two centres

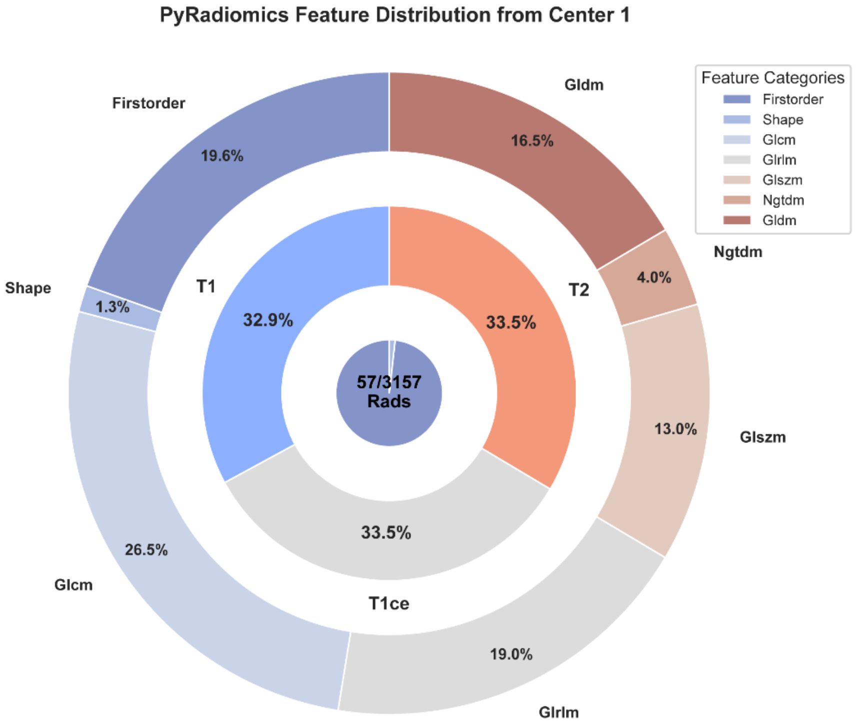

FigureS3: Distribution of the radiomics features extracted using 3D Regions of Interest (ROIs).

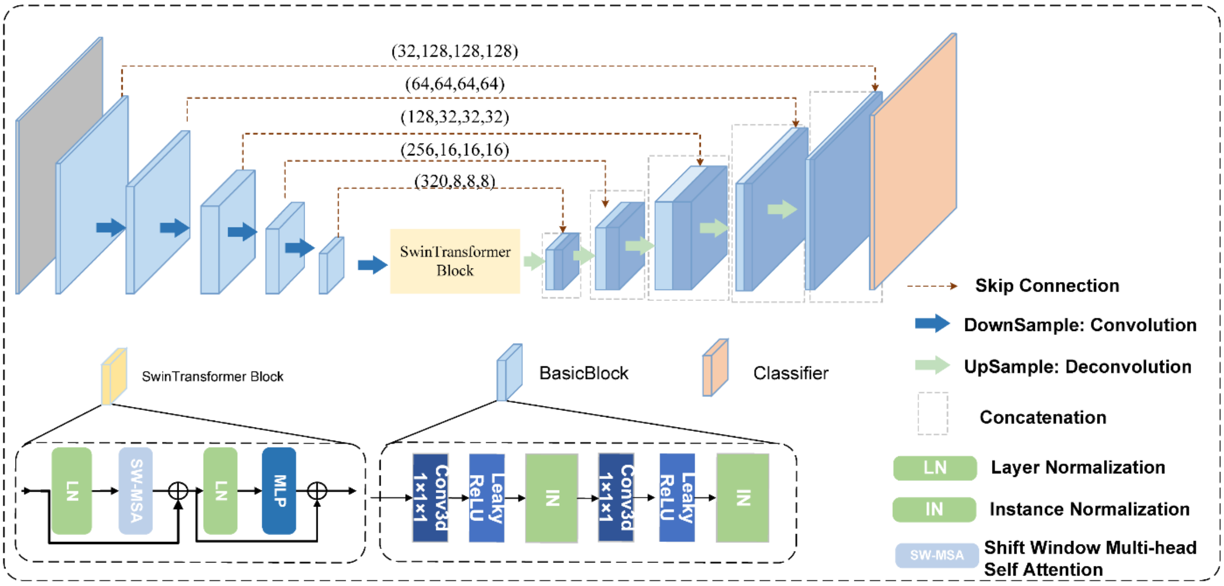

FigureS4: Architecture of the nnU-net with integrated multi-head attention mechanism. In the lower resolution path, Swin Transformer blocks are added, incorporating Shift Window Multi-Head Self Attention (SW-MSA) to enhance feature extraction.

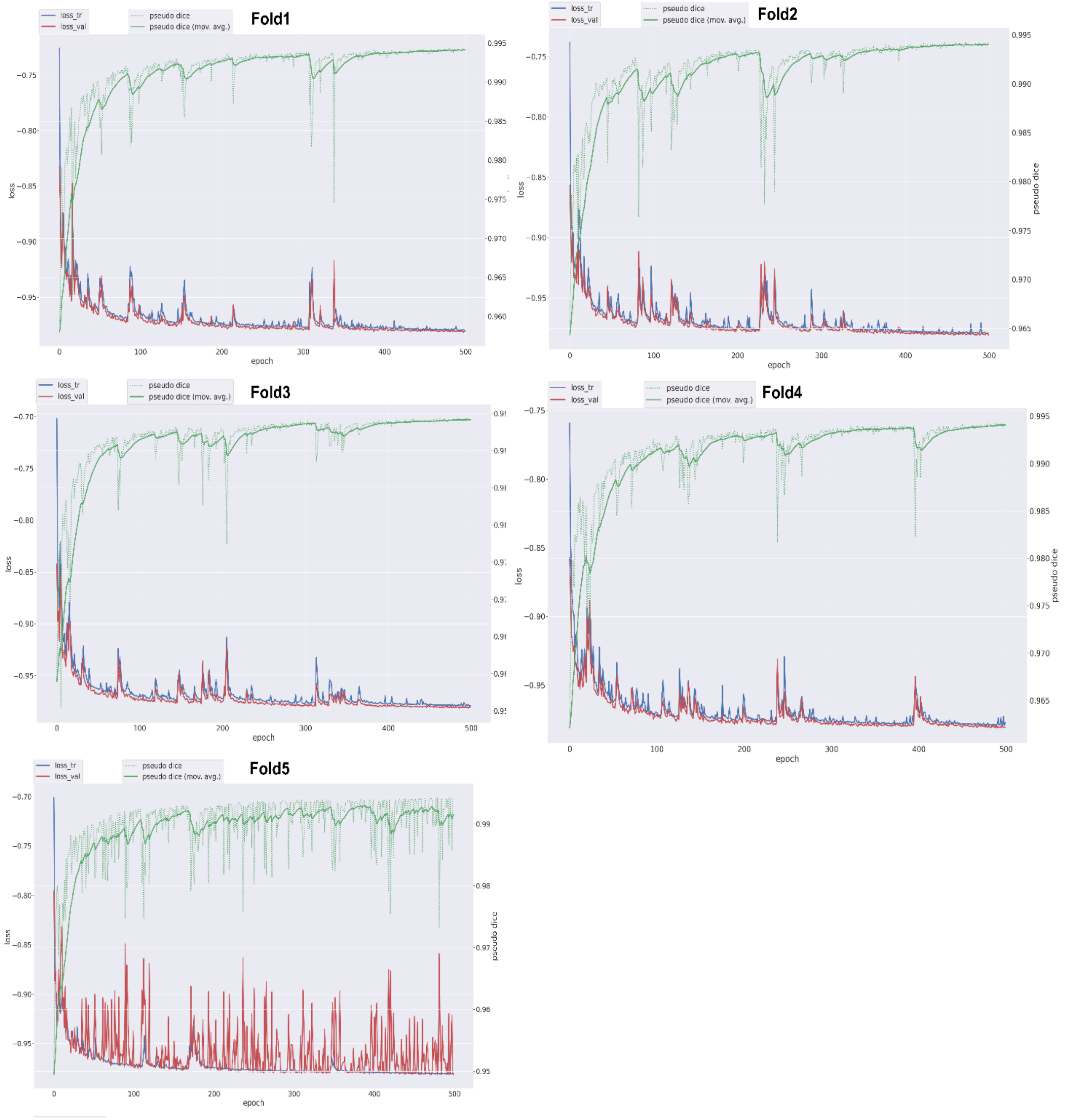

FigureS5: Learning Curves for 5-Fold Cross-Validation of nnU-Net in Brain Parenchyma Segmentation.

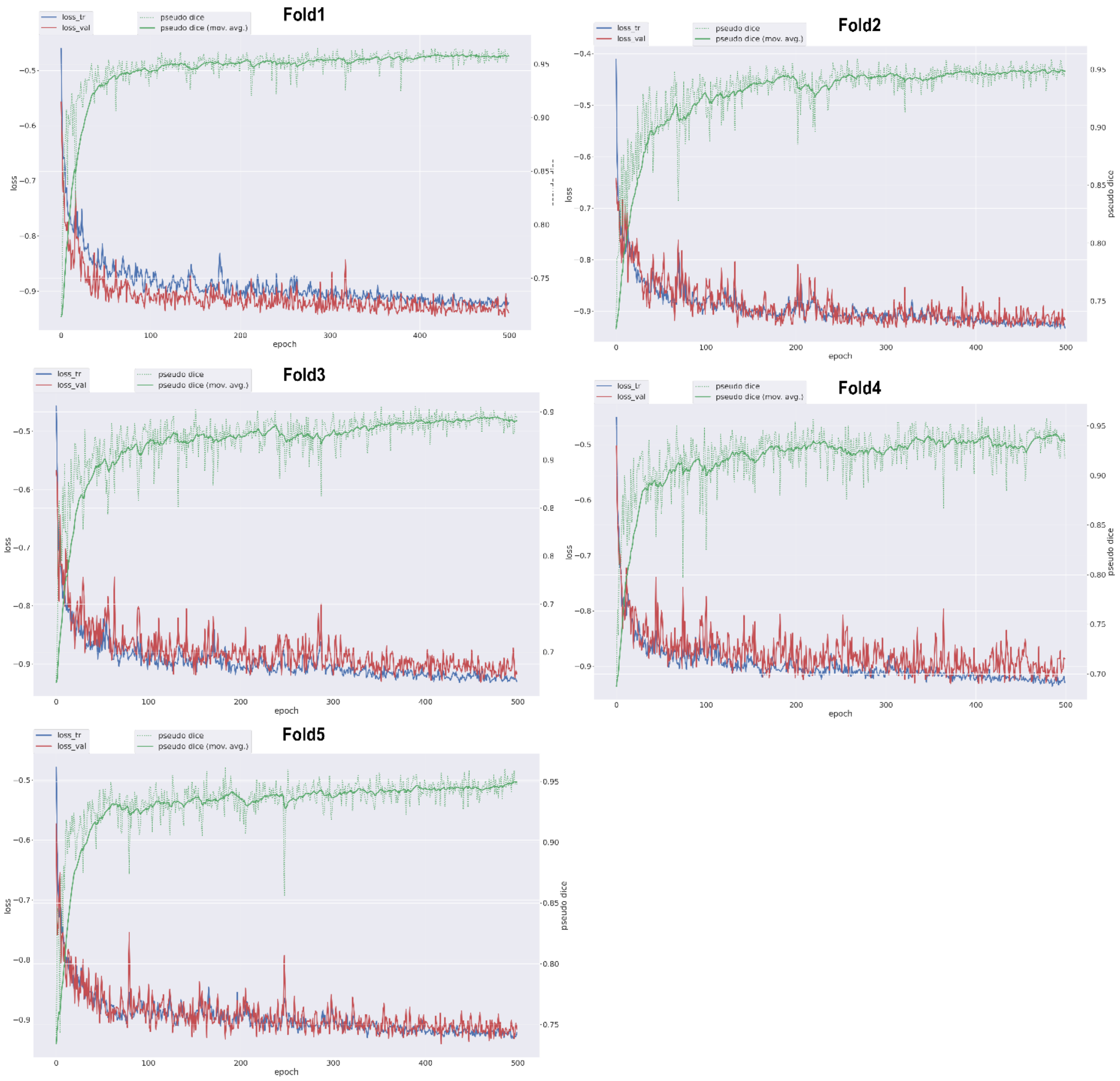

FigureS6： Learning Curves for 5-Fold Cross-Validation of nnU-Net in Tumor Segmentation.

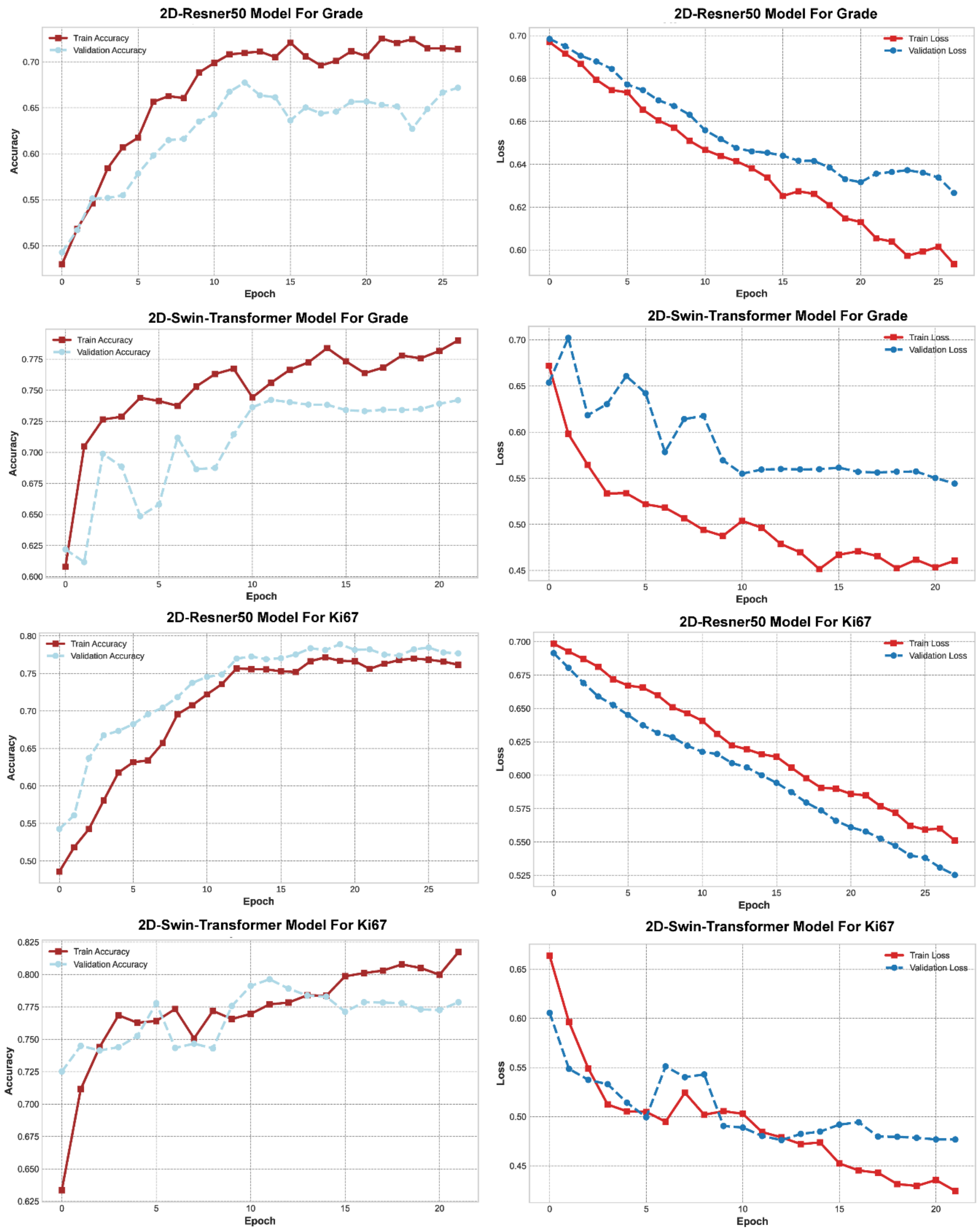

FigureS7: Average Learning Curve for 5-Fold Cross-Validation of 2D Deep Learning.

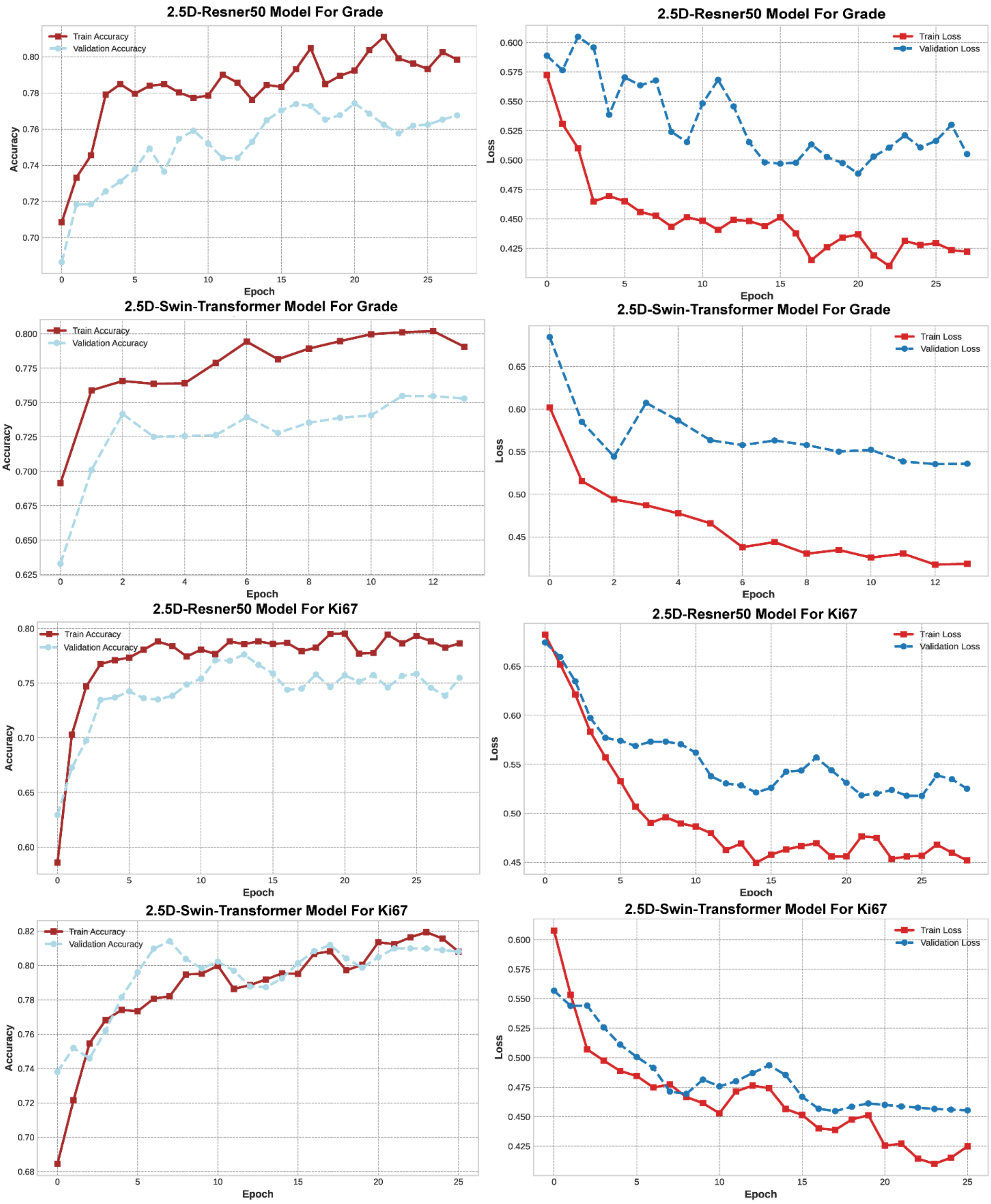

FigureS8: Average Learning Curve for 5-Fold Cross-Validation of 2.5D Deep Learning.

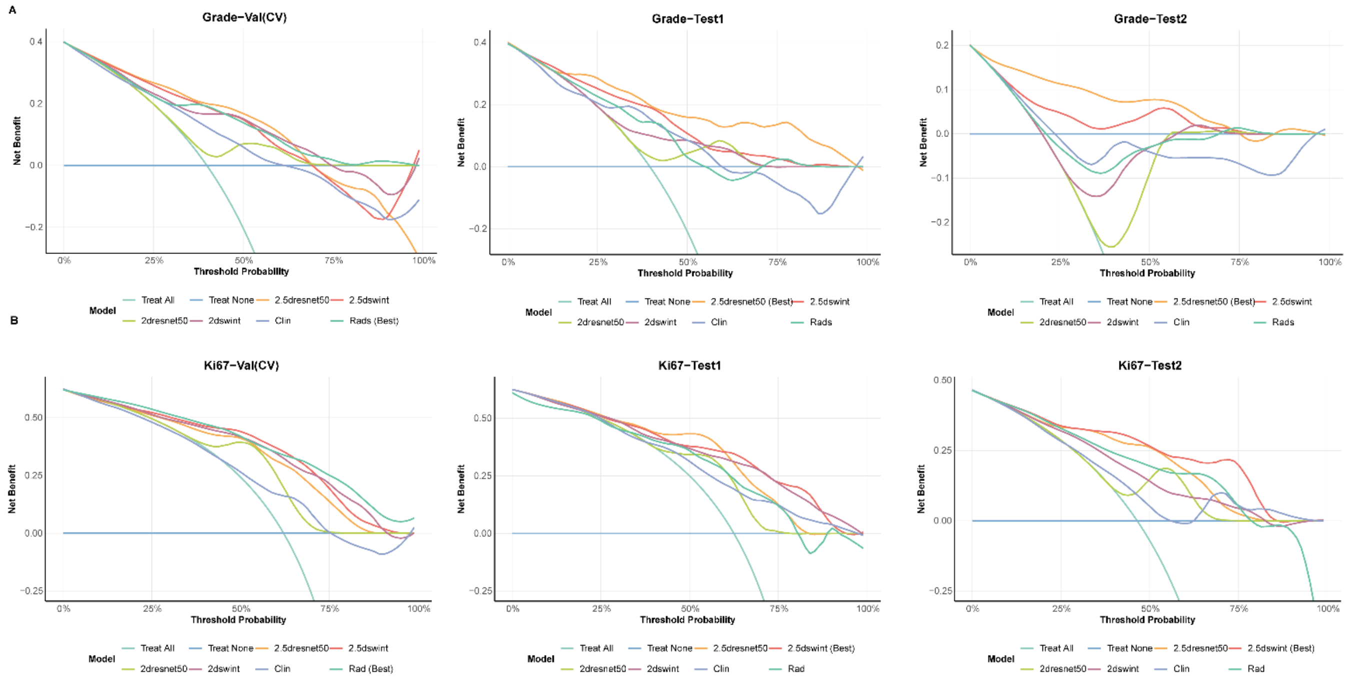

FigureS9: Decision Curve Analysis (DCA) for predicting meningioma outcomes: (A) Grade and (B) Ki67 expression. The curves illustrate the net benefit across various threshold probabilities for six models: 2.5D ResNet50, 2.5D Swin-Transformer, 2D ResNet50, 2D Swin-Transformer, Clinical (Clin), and Pyradiomics (Rads). The "Treat All" and "Treat None" strategies are included as benchmarks. In each panel, the model with the highest mean net benefit across the thresholds is marked as "Best" in the legend, highlighting its superior performance。

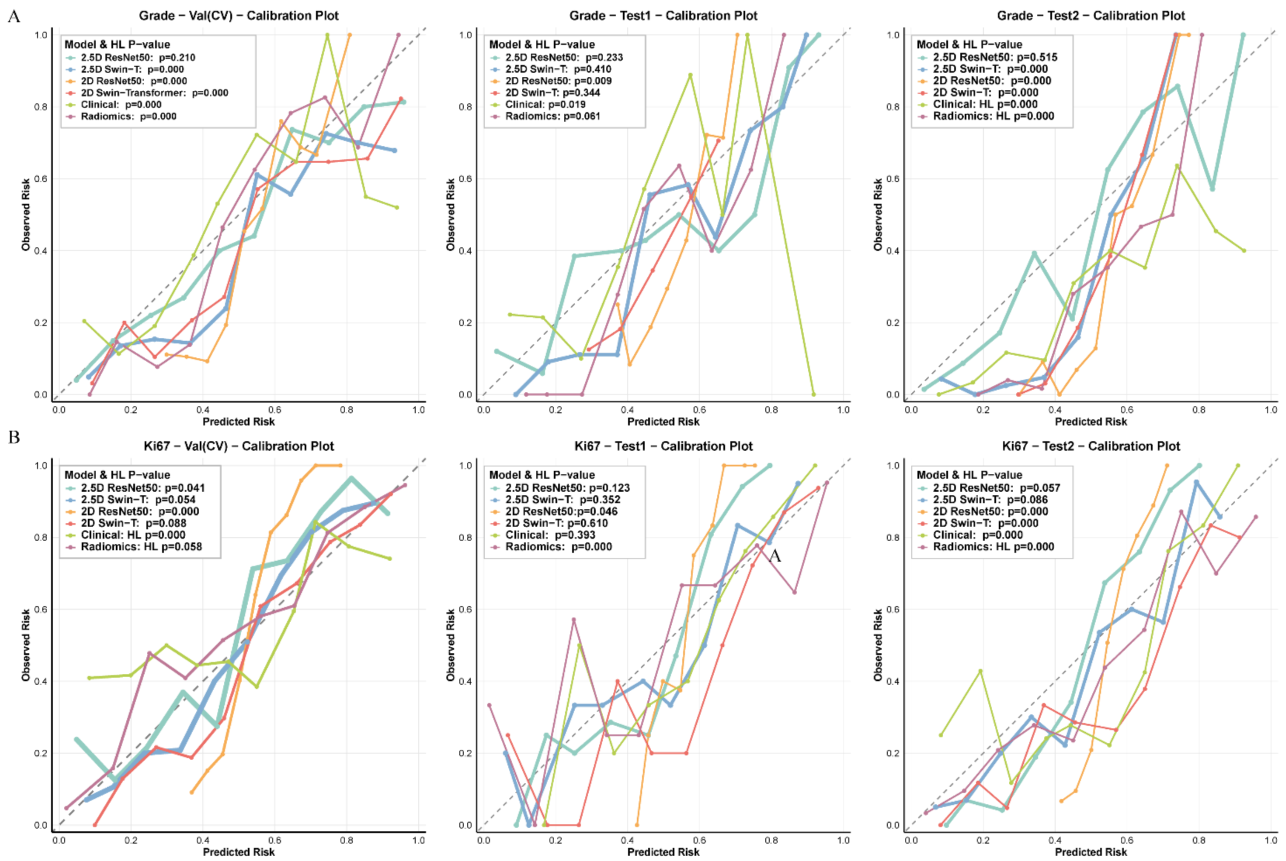

FigureS10: Calibration curves for Grade and Ki67 prediction models across datasets.(A) Grade prediction in Test1, Validation (CV), and Test2 datasets.(B) Ki67 prediction in Validation (CV), Test1, and Test2 datasets.Six models are compared: 2.5D ResNet50, 2D ResNet50, Clinical, 2.5D Swin-Transformer, 2D Swin-Transformer, and Radiomics. The diagonal dashed line represents perfect calibration, with curves closer to this line indicating better calibration performance. The Hosmer-Lemeshow (HL) test p-value, displayed in the top-left corner of each plot, provides a quantitative assessment of overall calibration goodness-of-fit; a p-value > 0.05 typically indicates acceptable calibration (no significant difference between predicted probabilities and observed frequencies). This figure demonstrates the reliability of each model's probability estimates across different datasets.

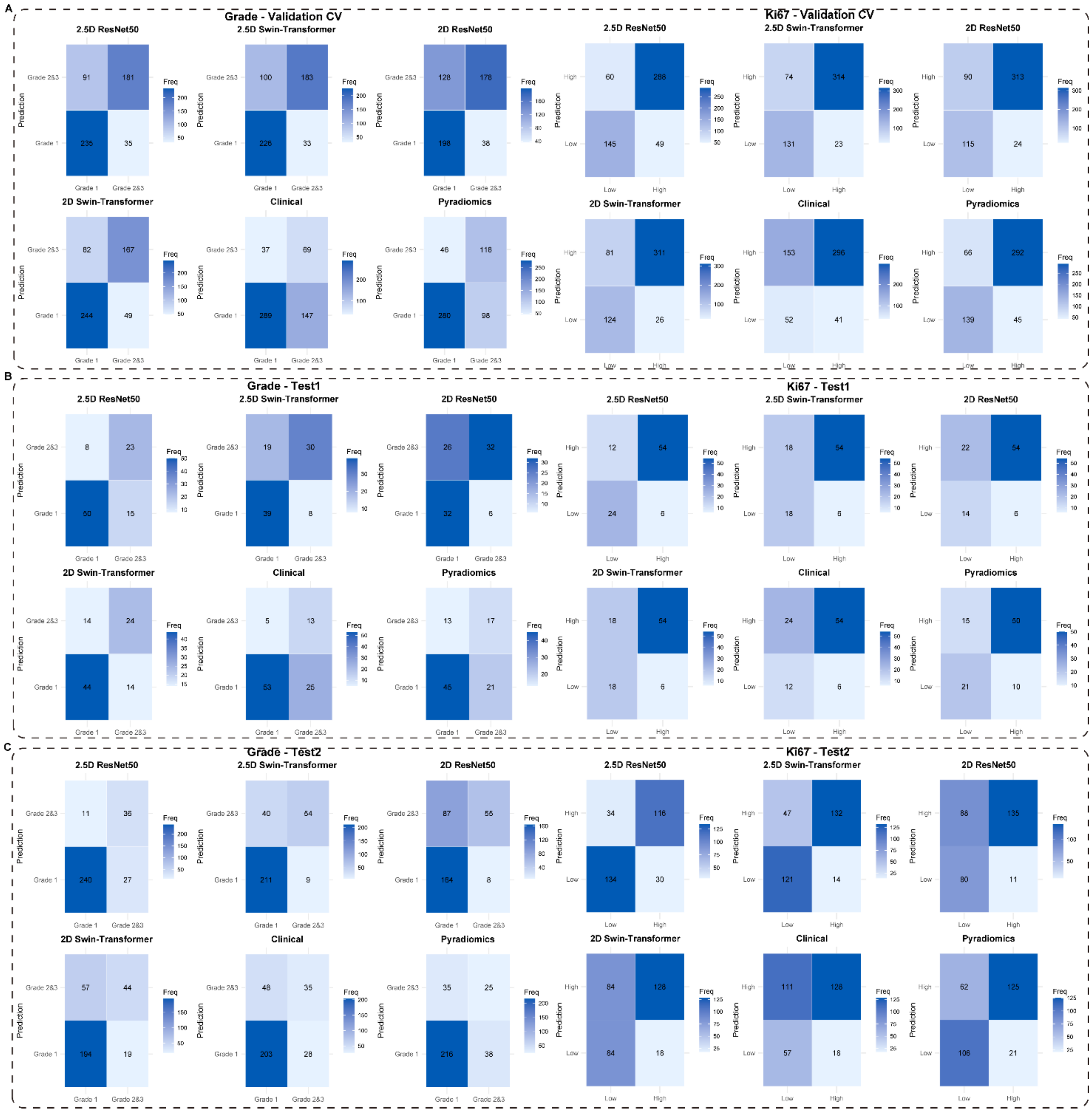

FigureS11: Confusion matrices for Grade and Ki67 prediction across three datasets (A: Validation CV, B: Test1, C: Test2). Each row shows results for both tasks using six models: 2.5D ResNet50, 2.5D Swin-Transformer, 2D ResNet50, 2D Swin-Transformer, Clinical, and Pyradiomics. Grade predictions are I/II vs. III, Ki67 are Low vs. High. Color intensity indicates case count, with diagonal elements showing correct predictions. This visualization compares model performance in predicting meningioma grade and Ki67 index across datasets and models.

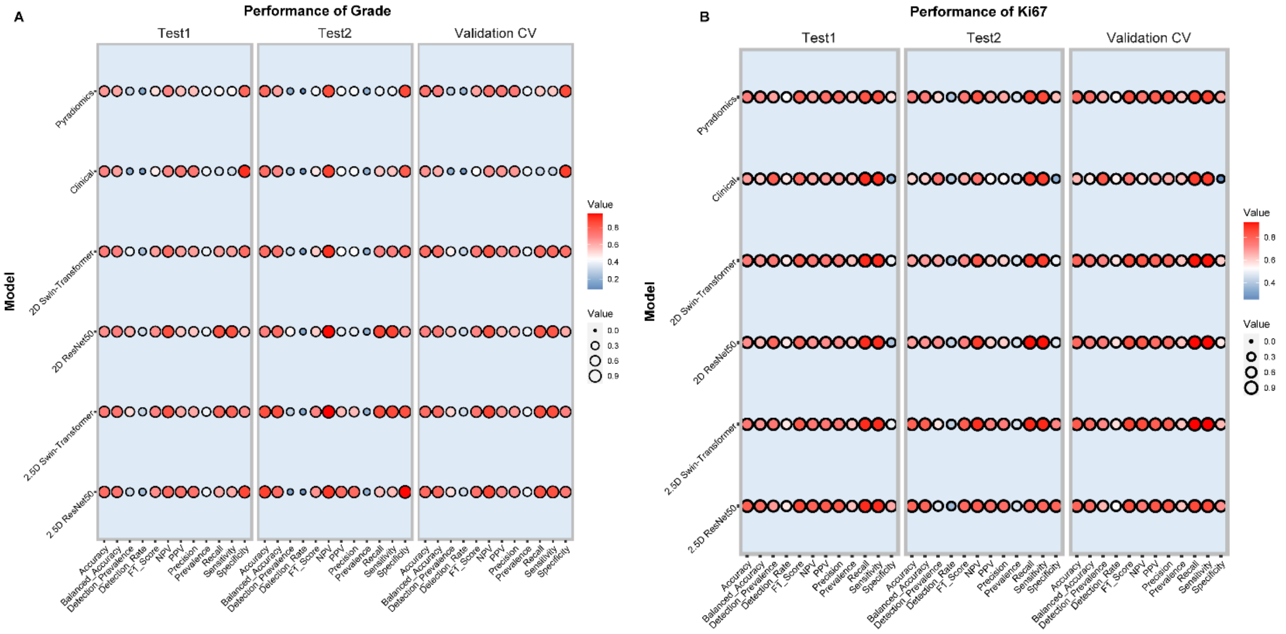

FigureS12: Bubble plots illustrating model performance across three datasets: Test1, Test2, and Validation (CV)..**(A)** Performance of Grade prediction models. Each bubble represents a performance metric, with bubble size and color indicating the value of the metric. Larger, more intensely colored bubbles signify higher performance values.**(B)** Performance of Ki67 prediction models, assessed similarly. Models include 2.5D ResNet50, 2D ResNet50, Clinical, 2.5D Swin-Transformer, 2D Swin-Transformer, and Pyradiomics. Metrics evaluated include accuracy, balanced accuracy, detection rate, detection prevalence, F1 score, PPV (Positive Predictive Value), NPV (Negative Predictive Value), sensitivity, and specificity. These plots provide a comprehensive comparison of each model's performance across different datasets for both Grade and Ki67 prediction tasks.

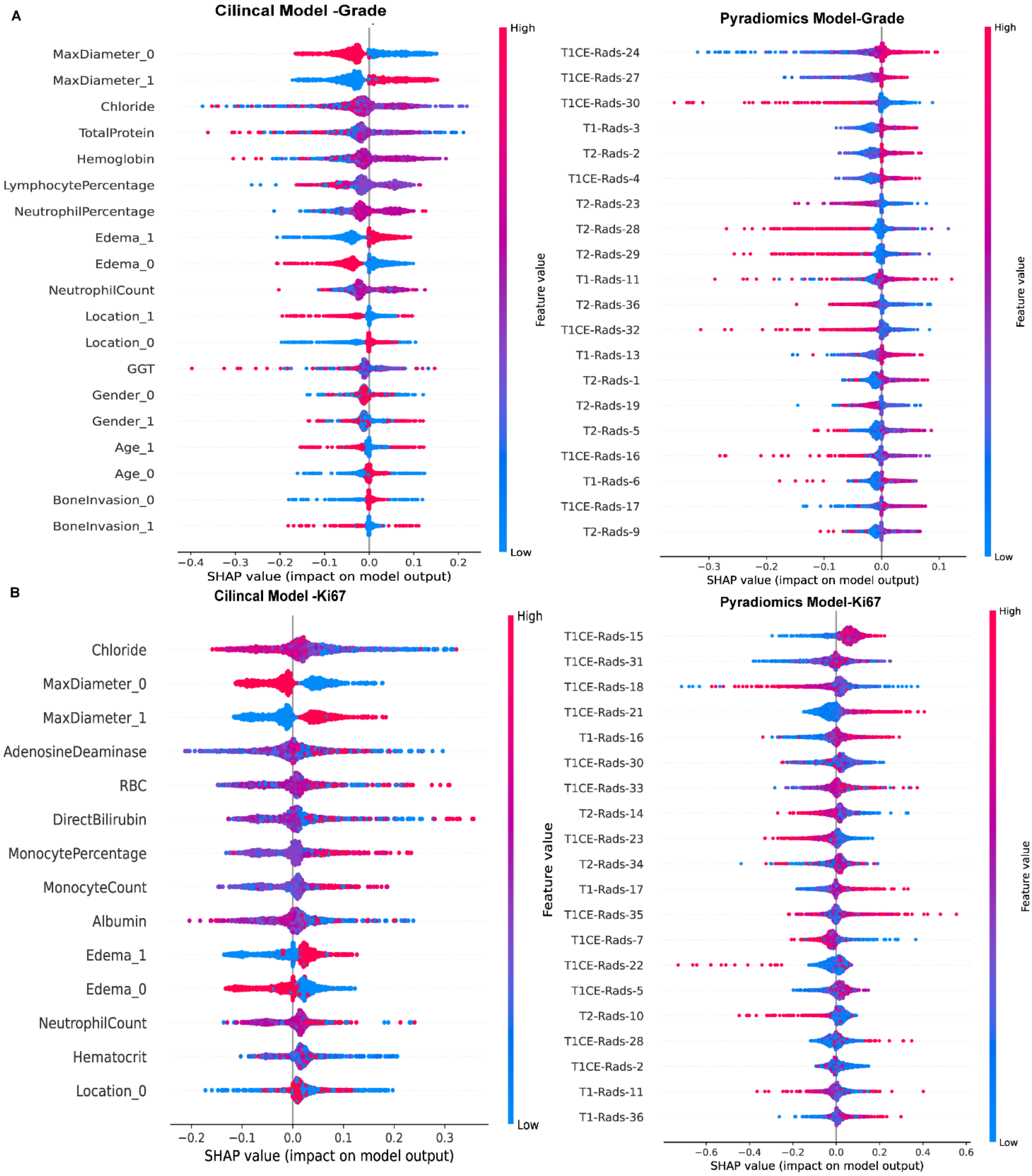

FigureS13: SHAP (SHapley Additive exPlanations) value plots are utilized to elucidate the contribution of each feature to the predictions made by machine learning models. The horizontal axis represents SHAP values, which quantify the impact of each feature on the prediction outcome; positive values suggest an increased likelihood of a high-risk prediction (e.g., higher WHO grade or Ki67 expression), whereas negative values indicate a reduced likelihood. The color gradient denotes the magnitude of feature values, with red representing higher values and blue indicating lower values. (A) The plots present SHAP value analyses for clinical and radiomics models regarding WHO grade prediction. (B) The plots present SHAP value analyses for clinical and radiomics models concerning Ki67 prediction.

Supplementary Tables

Manufacturer-Model Name	Counts
SIEMENS-Verio	232
SIEMENS-TrioTim	112
SIEMENS-Skyra	52
UIH-uMR770	66
Philips-ingenia CX	61
GE-DISCOVERY MR750w	20

Table S1：Distribution of Patient Counts Across Different MRI System Models of center1

	TR(ms)	TE(ms)	Slice Thickness (mm)	Matrix Size(mm)
T1WI	150 - 2938	2.48 - 27.264	4-5	(256 ~ 640) × (240 ~ 556)
T2WI	2817 - 7567	91 - 167.936	5	(320 ~ 768) × (320 ~ 668)
T1CE	6.5 - 1600	2.1 - 4.92	0.6-5	(256 ~ 1024) × (208 ~ 1024)

Table S2：Scan parameters of different sequences of center1.

Manufacturer-Model Name	Counts
SIEMENS-Prisma	156
SIEMENS-Verio	138
GE-DISCOVERY MR750w	20

Table S3：Distribution of Patient Counts Across Different MRI System Models of center2

	TR(ms)	TE(ms)	Slice Thickness (mm)	Matrix Size(mm)
T1WI	1750 - 2500	24.2 - 28.2	6	(384 ~ 512) × (384 ~ 512)
T2WI	3330 - 7710	104 - 126	6	(384 ~ 512) × (384 ~ 512)
T1CE	2100 - 2500	25 - 28.2	6	(384 ~ 512) × (384 ~ 512))

Table S4：Scan parameters of different sequences of center2.

Feature ID	Feature description
T2-Rads-1	gradient_ngtdm_Busyness
T2-Rads-2	original_shape_Maximum2DDiameterSlice
T1-Rads-3	original_shape_Maximum2DDiameterSlice
T1CE-Rads-4	original_shape_Maximum2DDiameterSlice
T2-Rads-5	original_ngtdm_Busyness
T1-Rads-6	log-sigma-3-mm-3D_glrlm_RunLengthNonUniformity
T1-Rads-7	square_glszm_GrayLevelNonUniformity
T1-Rads-8	original_glszm_GrayLevelNonUniformity
T2-Rads-9	wavelet-LHL_glszm_GrayLevelNonUniformity
T1-Rads-10	wavelet-LHL_glrlm_RunVariance
T1-Rads-11	wavelet-LLH_glrlm_RunVariance
T2-Rads-12	wavelet-HHL_ngtdm_Busyness
T1-Rads-13	wavelet-HLH_glrlm_RunVariance

T1CE-Rads-14	wavelet-LHL_glcm_Imc2
T1CE-Rads-15	log-sigma-5-mm-3D_firstorder_Kurtosis
T1CE-Rads-16	gradient_glrlm_GrayLevelNonUniformityNormalized
T1CE-Rads-17	gradient_glrlm_ShortRunLowGrayLevelEmphasis
T1CE-Rads-18	wavelet-LHH_gldm_DependenceNonUniformityNormalized
T2-Rads-19	gradient_glrlm_ShortRunEmphasis
T1-Rads-20	wavelet-HLL_glcm_Idn
T1CE-Rads-21	square_gldm_LargeDependenceLowGrayLevelEmphasis
T1-Rads-22	wavelet-HLL_gldm_LargeDependenceEmphasis
T2-Rads-23	wavelet-LHL_gldm_DependenceEntropy
T1CE-Rads-24	squareroot_gldm_DependenceEntropy
T2-Rads-25	wavelet-LLL_glcm_InverseVariance
T1-Rads-26	log-sigma-3-mm-3D_glcm_MaximumProbability
T1CE-Rads-27	log-sigma-3-mm-3D_glszm_ZoneEntropy
T2-Rads-28	wavelet-LLH_firstorder_Energy
T2-Rads-29	wavelet-LLH_firstorder_TotalEnergy
T1CE-Rads-30	log-sigma-5-mm-3D_gldm_DependenceNonUniformityNormalized
T1-Rads-31	wavelet-HLH_firstorder_Maximum
T1CE-Rads-32	square_glrlm_LongRunHighGrayLevelEmphasis
T1CE-Rads-33	gradient_firstorder_Maximum
T2-Rads-34	exponential_glszm_GrayLevelNonUniformity
T1CE-Rads-35	squareroot_gldm_DependenceVariance
T2-Rads-36	square_glszm_GrayLevelVariance
T2-Rads-37	log-sigma-3-mm-3D_firstorder_Kurtosis
T2-Rads-38	log-sigma-5-mm-3D_glcm_ClusterShade
T2-Rads-39	wavelet-HHL_glszm_GrayLevelVariance
T2-Rads-40	log-sigma-3-mm-3D_firstorder_Skewness
T1CE-Rads-41	diagnostics_Image-interpolated_Mean
T2-Rads-42	wavelet-HLL_glszm_GrayLevelVariance
T2-Rads-43	square_firstorder_Kurtosis

Table S5： Descriptions of the key radiomics features for predicting grade.

**Feature ID**	**Feature description**
T1-Rads-1	original_shape_Maximum2DDiameterSlice
T1CE-Rads-2	wavelet-LLH_glrlm_RunLengthNonUniformity
T2-Rads-3	original_shape_MajorAxisLength
T2-Rads-4	wavelet-LLL_ngtdm_Strength
T1CE-Rads-5	log-sigma-3-mm-3D_glcm_Imc1
T1CE-Rads-6	gradient_ngtdm_Strength
T1CE-Rads-7	log-sigma-3-mm-3D_glcm_Imc2
T2-Rads-8	log-sigma-3-mm-3D_glrlm_ShortRunEmphasis
T1-Rads-9	gradient_ngtdm_Busyness
T2-Rads-10	wavelet-HLL_ngtdm_Strength
T1-Rads-11	wavelet-LLH_glrlm_RunVariance

T2-Rads-12	log-sigma-3-mm-3D_glcm_DifferenceVariance
T1-Rads-13	log-sigma-5-mm-3D_glcm_Imc2
T2-Rads-14	gradient_glrlm_ShortRunEmphasis
T1CE-Rads-15	log-sigma-5-mm-3D_gldm_DependenceVariance
T1-Rads-16	wavelet-HLH_glrlm_RunVariance
T1-Rads-17	wavelet-LHH_glrlm_RunVariance
T1CE-Rads-18	lbp-2D_firstorder_Skewness
T2-Rads-19	logarithm_glrlm_ShortRunEmphasis
T2-Rads-20	wavelet-LLL_glcm_InverseVariance
T1CE-Rads-21	log-sigma-5-mm-3D_gldm_DependenceNonUniformityNormalized
T1CE-Rads-22	log-sigma-5-mm-3D_gldm_SmallDependenceHighGrayLevelEmphasis
T1CE-Rads-23	gradient_glrlm_ShortRunLowGrayLevelEmphasis
T1-Rads-24	wavelet-HHH_firstorder_Energy
T1-Rads-25	wavelet-HHH_firstorder_TotalEnergy
T1-Rads-26	wavelet-HLL_glrlm_LowGrayLevelRunEmphasis
T1CE-Rads-27	wavelet-LHL_glcm_Imc1
T1CE-Rads-28	square_glszm_SizeZoneNonUniformity
T1-Rads-29	square_gldm_DependenceVariance
T1CE-Rads-30	log-sigma-3-mm-3D_glszm_SmallAreaHighGrayLevelEmphasis
T1CE-Rads-31	wavelet-LLH_glcm_InverseVariance
T1-Rads-32	log-sigma-5-mm-3D_firstorder_Uniformity
T1CE-Rads-33	logarithm_gldm_DependenceEntropy
T2-Rads-34	wavelet-LLH_glcm_Imc1
T1CE-Rads-35	wavelet-LHL_gldm_DependenceVariance
T1-Rads-36	wavelet-HLH_glrlm_RunEntropy

Table S6： Descriptions of the key radiomics features for predicting ki67.

	Model and Stage (WHO grade)		Accuracy	Sensitivity	Specificity	AUC	p value1	p value2
Internal Testing set (n=96)	Gender: Female (n=68)	2.5D ResNet50	**0.76 [0.66, 0.85]**	0.56 [0.36, 0.76]	0.88 [0.78, 0.98]	**0.87 [0.78, 0.95]**	reference	0.184
		2.5D Swin-T	0.72 [0.60, 0.81]	0.80 [0.64, 0.95]	0.67 [0.52, 0.80]	0.82 [0.71, 0.90]	0.184	reference
		2D ResNet50	0.71 [0.60, 0.81]	0.88 [0.74, 1.00]	0.60 [0.45, 0.75]	0.80 [0.68, 0.90]	0.167	0.706
		2D Swin-T	0.72 [0.62, 0.82]	0.56 [0.37, 0.76]	0.81 [0.69, 0.93]	0.76 [0.63, 0.87]	0.017*	0.172
		Clinical	0.69 [0.57, 0.79]	0.32 [0.15, 0.52]	0.91 [0.81, 0.98]	0.69 [0.55, 0.83]	0.012*	0.104
		Radiomics	0.62 [0.50, 0.72]	0.32 [0.14, 0.50]	0.79 [0.66, 0.90]	0.70 [0.56, 0.81]	0.004*	0.014*
	Gender: Male (n=28)	2.5D ResNet50	**0.75 [0.57, 0.89]**	0.69 [0.43, 0.92]	0.80 [0.57, 1.00]	**0.83 [0.67, 0.97]**	reference	0.526
		2.5D Swin-T	0.71 [0.57, 0.86]	0.77 [0.53, 1.00]	0.67 [0.42, 0.90]	0.80 [0.60, 0.95]	0.526	reference
		2D ResNet50	0.57 [0.39, 0.75]	0.77 [0.53, 1.00]	0.40 [0.15, 0.67]	0.70 [0.48, 0.89]	0.042*	0.300
		2D Swin-T	0.68 [0.50, 0.86]	0.77 [0.50, 1.00]	0.60 [0.33, 0.85]	0.71 [0.49, 0.90]	0.025*	0.214
		Clinical	0.68 [0.50, 0.86]	0.38 [0.12, 0.67]	0.93 [0.78, 1.00]	0.78 [0.57, 0.95]	0.699	0.900
		Radiomics	0.71 [0.54, 0.86]	0.69 [0.42, 0.93]	0.73 [0.50, 0.93]	0.81 [0.64, 0.95]	0.774	0.818
	Age: ≤65 years (n=75)	2.5D ResNet50	**0.80 [0.71, 0.88]**	0.57 [0.38, 0.75]	0.94 [0.86, 1.00]	**0.86 [0.76, 0.94]**	reference	0.229
		2.5D Swin-T	0.73 [0.64, 0.83]	0.75 [0.57, 0.90]	0.72 [0.60, 0.84]	0.81 [0.72, 0.91]	0.229	reference
		2D ResNet50	0.69 [0.59, 0.79]	0.82 [0.67, 0.96]	0.62 [0.47, 0.74]	0.77 [0.64, 0.88]	0.045*	0.320
		2D Swin-T	0.72 [0.63, 0.83]	0.61 [0.43, 0.79]	0.79 [0.67, 0.90]	0.75 [0.63, 0.86]	0.002*	0.070
		Clinical	0.71 [0.60, 0.81]	0.29 [0.12, 0.47]	0.96 [0.89, 1.00]	0.72 [0.58, 0.85]	0.078	0.247
		Radiomics	0.63 [0.52, 0.75]	0.32 [0.16, 0.52]	0.81 [0.69, 0.92]	0.77 [0.66, 0.88]	0.115	0.307
	Age: >65 years (n=21)	2.5D ResNet50	0.62 [0.38, 0.81]	0.70 [0.38, 1.00]	0.54 [0.22, 0.85]	**0.81 [0.59, 0.96]**	reference	0.726
		2.5D Swin-T	0.67 [0.48, 0.86]	0.90 [0.64, 1.00]	0.46 [0.15, 0.79]	0.78 [0.53, 0.96]	0.726	reference
		2D ResNet50	0.57 [0.33, 0.76]	0.90 [0.67, 1.00]	0.27 [0.00, 0.57]	0.78 [0.53, 0.96]	0.805	1.000
		2D Swin-T	0.67 [0.48, 0.86]	0.70 [0.36, 1.00]	0.64 [0.33, 0.92]	0.74 [0.51, 0.94]	0.580	0.686
		Clinical	0.62 [0.38, 0.81]	0.50 [0.18, 0.82]	0.73 [0.44, 1.00]	0.66 [0.38, 0.91]	0.343	0.436
		Radiomics	**0.71 [0.52, 0.90]**	0.80 [0.50, 1.00]	0.64 [0.33, 0.90]	0.70 [0.44, 0.92]	0.336	0.507

Table S7. Stratified Performance comparison of the models in predicting WHO grade in the Internal Testing set

	Model and Stage (WHO grade)		Accuracy	Sensitivity	Specificity	AUC	p value1	p value2
External testing set (n=314)	Gender: Female (n=234)	2.5D ResNet50	**0.88 [0.83, 0.92]**	0.50 [0.32, 0.66]	0.95 [0.92, 0.98]	**0.88 [0.82, 0.93]**	reference	0.934
		2.5D Swin-T	0.85 [0.80, 0.89]	0.82 [0.70, 0.94]	0.85 [0.80, 0.90]	**0.88 [0.80, 0.93]**	0.934	reference
		2D ResNet50	0.70 [0.64, 0.77]	0.82 [0.70, 0.94]	0.68 [0.61, 0.75]	0.81 [0.72, 0.88]	0.036*	0.030*
		2D Swin-T	0.75 [0.70, 0.81]	0.60 [0.46, 0.76]	0.78 [0.72, 0.84]	0.81 [0.74, 0.87]	0.012*	0.010*
		Clinical	0.76 [0.71, 0.82]	0.60 [0.43, 0.75]	0.80 [0.74, 0.85]	0.77 [0.68, 0.85]	0.016*	0.022*
		Radiomics	0.77 [0.72, 0.82]	0.35 [0.20, 0.51]	0.86 [0.81, 0.91]	0.78 [0.71, 0.85]	0.003*	0.008*
	Gender: Male (n=80)	2.5D ResNet50	**0.89 [0.81, 0.95]**	0.70 [0.47, 0.88]	0.96 [0.91, 1.00]	**0.94 [0.87, 0.98]**	reference	0.883
		2.5D Swin-T	0.84 [0.75, 0.91]	0.91 [0.79, 1.00]	0.81 [0.70, 0.91]	0.93 [0.87, 0.98]	0.883	reference
		2D ResNet50	0.68 [0.56, 0.78]	0.96 [0.85, 1.00]	0.56 [0.43, 0.68]	0.92 [0.84, 0.98]	0.646	0.753
		2D Swin-T	0.78 [0.68, 0.86]	0.87 [0.71, 1.00]	0.74 [0.62, 0.85]	0.90 [0.82, 0.96]	0.368	0.345
		Clinical	0.74 [0.64, 0.82]	0.48 [0.26, 0.69]	0.84 [0.73, 0.93]	0.77 [0.65, 0.87]	0.006*	0.001*
		Radiomics	0.75 [0.65, 0.85]	0.48 [0.29, 0.70]	0.86 [0.76, 0.95]	0.83 [0.74, 0.91]	0.039*	0.030*
	Age: ≤65 years (n=222)	2.5D ResNet50	**0.90 [0.85, 0.94]**	0.61 [0.46, 0.76]	0.96 [0.93, 0.98]	**0.90 [0.84, 0.94]**	reference	0.836
		2.5D Swin-T	0.86 [0.81, 0.90]	0.88 [0.76, 0.97]	0.86 [0.81, 0.91]	0.89 [0.82, 0.95]	0.836	reference
		2D ResNet50	0.72 [0.67, 0.78]	0.90 [0.81, 0.98]	0.68 [0.61, 0.75]	0.86 [0.80, 0.92]	0.197	0.255
		2D Swin-T	0.78 [0.72, 0.83]	0.68 [0.53, 0.82]	0.80 [0.73, 0.85]	0.85 [0.79, 0.90]	0.052	0.075
		Clinical	0.76 [0.70, 0.81]	0.56 [0.41, 0.70]	0.81 [0.75, 0.86]	0.76 [0.67, 0.83]	0.003*	0.005*
		Radiomics	0.78 [0.73, 0.84]	0.39 [0.25, 0.54]	0.87 [0.82, 0.92]	0.81 [0.75, 0.87]	0.003*	0.007*
	Age: >65 years (n=92)	2.5D ResNet50	**0.84 [0.76, 0.91]**	0.50 [0.29, 0.71]	0.94 [0.88, 0.99]	**0.89 [0.82, 0.95]**	reference	0.939
		2.5D Swin-T	0.80 [0.72, 0.88]	0.82 [0.65, 0.96]	0.80 [0.71, 0.89]	**0.89 [0.81, 0.95]**	0.939	reference
		2D ResNet50	0.63 [0.53, 0.73]	0.82 [0.65, 0.96]	0.57 [0.46, 0.69]	0.79 [0.66, 0.90]	0.056	0.058
		2D Swin-T	0.72 [0.62, 0.80]	0.73 [0.50, 0.91]	0.71 [0.61, 0.82]	0.81 [0.71, 0.90]	0.096	0.058
		Clinical	0.75 [0.66, 0.84]	0.54 [0.33, 0.75]	0.81 [0.72, 0.90]	0.78 [0.66, 0.88]	0.105	0.071
		Radiomics	0.73 [0.64, 0.81]	0.41 [0.19, 0.61]	0.83 [0.74, 0.91]	0.78 [0.69, 0.88]	0.043*	0.037*

Table S8. Performance comparison of the models in predicting the WHO grade in the External Testing set

	Model and Stage (Ki-67)		Accuracy	Sensitivity	Specificity	AUC	p value1	p value2
Internal Testing set (n=96)	Gender: Female (n=68)	2.5D ResNet50	**0.81 [0.72, 0.90]**	0.92 [0.83, 1.00]	0.66 [0.47, 0.83]	**0.88 [0.79, 0.95]**	reference	0.325
		2.5D Swin-T	0.72 [0.60, 0.82]	0.90 [0.80, 0.98]	0.48 [0.29, 0.65]	0.84 [0.72, 0.92]	0.325	reference
		2D ResNet50	0.69 [0.57, 0.79]	0.92 [0.83, 1.00]	0.38 [0.20, 0.56]	0.83 [0.74, 0.92]	0.296	0.984
		2D Swin-T	0.75 [0.65, 0.85]	0.92 [0.83, 1.00]	0.52 [0.33, 0.71]	0.82 [0.71, 0.92]	0.280	0.791
		Clinical	0.66 [0.56, 0.78]	0.85 [0.72, 0.95]	0.41 [0.23, 0.61]	0.73 [0.60, 0.85]	0.014*	0.122
		Radiomics	0.72 [0.62, 0.82]	0.82 [0.70, 0.93]	0.59 [0.41, 0.75]	0.74 [0.62, 0.85]	0.006*	0.069
	Gender: Male (n=28)	2.5D ResNet50	**0.82 [0.68, 0.96]**	0.86 [0.70, 1.00]	0.71 [0.33, 1.00]	0.81 [0.55, 0.99]	reference	0.299
		2.5D Swin-T	**0.82 [0.68, 0.96]**	0.90 [0.76, 1.00]	0.57 [0.17, 1.00]	**0.86 [0.67, 1.00]**	0.299	reference
		2D ResNet50	0.75 [0.57, 0.89]	0.86 [0.67, 1.00]	0.43 [0.00, 0.83]	0.77 [0.56, 0.95]	0.671	0.250
		2D Swin-T	0.75 [0.61, 0.89]	0.86 [0.70, 1.00]	0.43 [0.00, 0.82]	0.86 [0.71, 0.98]	0.563	0.920
		Clinical	0.75 [0.57, 0.89]	1.00	0.00 [0.00, 0.00]	0.70 [0.41, 0.93]	0.425	0.219
		Radiomics	0.79 [0.64, 0.93]	0.86 [0.70, 1.00]	0.57 [0.17, 1.00]	0.78 [0.50, 0.98]	0.558	0.306
	Age: ≤65 years (n=75)	2.5D ResNet50	**0.81 [0.72, 0.89]**	0.86 [0.76, 0.96]	0.74 [0.58, 0.90]	**0.85 [0.76, 0.93]**	reference	0.517
		2.5D Swin-T	0.73 [0.63, 0.83]	0.86 [0.75, 0.95]	0.55 [0.38, 0.73]	0.83 [0.73, 0.91]	0.517	reference
		2D ResNet50	0.68 [0.56, 0.79]	0.89 [0.78, 0.98]	0.39 [0.21, 0.56]	0.82 [0.72, 0.90]	0.411	0.704
		2D Swin-T	0.73 [0.63, 0.83]	0.86 [0.75, 0.95]	0.55 [0.38, 0.72]	0.83 [0.73, 0.91]	0.616	0.933
		Clinical	0.64 [0.53, 0.75]	0.86 [0.75, 0.96]	0.32 [0.17, 0.48]	0.71 [0.59, 0.83]	0.029*	0.068
		Radiomics	0.71 [0.60, 0.81]	0.77 [0.65, 0.90]	0.61 [0.43, 0.78]	0.74 [0.63, 0.85]	0.021*	0.046*
	Age: >65 years (n=21)	2.5D ResNet50	0.81 [0.62, 0.95]	1.00	0.20 [0.00, 0.67]	**0.91 [0.70, 1.00]**	reference	0.819
		2.5D Swin-T	0.81 [0.62, 0.95]	1.00	0.20 [0.00, 0.67]	0.89 [0.70, 1.00]	0.819	reference
		2D ResNet50	0.81 [0.62, 0.95]	0.94 [0.80, 1.00]	0.40 [0.00, 1.00]	0.75 [0.42, 1.00]	0.040*	0.397
		2D Swin-T	0.81 [0.62, 0.95]	1.00	0.20 [0.00, 0.67]	0.78 [0.44, 1.00]	0.319	0.496
		Clinical	**0.86 [0.67, 1.00]**	1.00	0.40 [0.00, 1.00]	0.78 [0.50, 1.00]	0.257	0.514
		Radiomics	**0.86 [0.71, 1.00]**	1.00	0.40 [0.00, 1.00]	0.71 [0.41, 1.00]	0.087	0.341

Table S9. Stratified Performance comparison of the models in predicting the Ki-67 in the Internal Testing set

	Model and Stage (Ki-67)		Accuracy	Sensitivity	Specificity	AUC	p value1	p value2
External testing set (n=314)	Gender: Female (n=234)	2.5D ResNet50	0.80 [0.74, 0.85]	0.76 [0.67, 0.84]	0.83 [0.76, 0.89]	0.86 [0.81, 0.91]	reference	0.179
		2.5D Swin-T	0.78 [0.72, 0.83]	0.87 [0.80, 0.93]	0.71 [0.63, 0.79]	0.88 [0.84, 0.92]	0.179	reference
		2D ResNet50	0.66 [0.59, 0.72]	0.92 [0.87, 0.97]	0.46 [0.37, 0.56]	0.81 [0.76, 0.87]	0.042*	0.004*
		2D Swin-T	0.67 [0.60, 0.73]	0.87 [0.80, 0.93]	0.52 [0.43, 0.60]	0.81 [0.75, 0.86]	0.041*	0.002*
		Clinical	0.58 [0.53, 0.65]	0.86 [0.79, 0.92]	0.38 [0.30, 0.47]	0.76 [0.69, 0.82]	0.003*	<0.001*
		Radiomics	0.74 [0.68, 0.80]	0.86 [0.79, 0.92]	0.65 [0.57, 0.73]	0.81 [0.76, 0.87]	0.134	0.016*
	Gender: Male (n=80)	2.5D ResNet50	0.79 [0.69, 0.88]	0.87 [0.76, 0.96]	0.68 [0.52, 0.84]	0.90 [0.82, 0.95]	reference	0.643
		2.5D Swin-T	0.89 [0.81, 0.95]	0.98 [0.93, 1.00]	0.76 [0.61, 0.91]	0.91 [0.82, 0.98]	0.643	reference
		2D ResNet50	0.76 [0.65, 0.85]	0.94 [0.85, 1.00]	0.53 [0.34, 0.69]	0.84 [0.73, 0.92]	0.055	0.031*
		2D Swin-T	0.70 [0.60, 0.80]	0.89 [0.80, 0.98]	0.44 [0.26, 0.60]	0.78 [0.68, 0.88]	0.013*	0.004*
		Clinical	0.60 [0.49, 0.71]	0.91 [0.83, 0.98]	0.18 [0.06, 0.32]	0.74 [0.63, 0.85]	0.011*	0.008*
		Radiomics	0.72 [0.62, 0.82]	0.85 [0.75, 0.94]	0.56 [0.39, 0.72]	0.84 [0.74, 0.93]	0.217	0.111
	Age: ≤65 years (n=222)	2.5D ResNet50	0.80 [0.74, 0.85]	0.78 [0.70, 0.86]	0.81 [0.74, 0.88]	0.88 [0.83, 0.92]	reference	0.330
		2.5D Swin-T	0.81 [0.76, 0.86]	0.91 [0.85, 0.96]	0.73 [0.65, 0.81]	0.89 [0.85, 0.93]	0.330	reference
		2D ResNet50	0.68 [0.63, 0.75]	0.92 [0.86, 0.97]	0.49 [0.40, 0.58]	0.82 [0.76, 0.88]	0.015*	0.002*
		2D Swin-T	0.65 [0.58, 0.71]	0.86 [0.79, 0.92]	0.47 [0.39, 0.56]	0.79 [0.73, 0.84]	<0.001*	<0.001*
		Clinical	0.57 [0.50, 0.64]	0.89 [0.83, 0.95]	0.30 [0.22, 0.39]	0.75 [0.69, 0.82]	<0.001*	<0.001*
		Radiomics	0.76 [0.70, 0.81]	0.85 [0.77, 0.92]	0.68 [0.60, 0.76]	0.82 [0.77, 0.88]	0.078	0.013*
	Age: >65 years (n=92)	2.5D ResNet50	0.79 [0.71, 0.87]	0.83 [0.72, 0.92]	0.76 [0.62, 0.88]	0.85 [0.76, 0.92]	reference	0.110
		2.5D Swin-T	0.79 [0.71, 0.87]	0.89 [0.80, 0.98]	0.70 [0.56, 0.82]	0.89 [0.83, 0.95]	0.110	reference
		2D ResNet50	0.68 [0.59, 0.78]	0.94 [0.85, 1.00]	0.43 [0.29, 0.58]	0.82 [0.72, 0.90]	0.421	0.040*
		2D Swin-T	0.74 [0.65, 0.84]	0.91 [0.82, 0.98]	0.56 [0.43, 0.71]	0.85 [0.77, 0.93]	0.847	0.335
		Clinical	0.64 [0.54, 0.74]	0.85 [0.74, 0.94]	0.43 [0.30, 0.59]	0.77 [0.66, 0.87]	0.177	0.014*
		Radiomics	0.68 [0.59, 0.78]	0.87 [0.77, 0.96]	0.50 [0.34, 0.65]	0.80 [0.70, 0.89]	0.435	0.053

Table S10. Stratified Performance comparison of the models in predicting the Ki-67 in the External Testing set

Supplementary Methods

1. ANTs Registration Parameters

Image registration to the MNI template was performed using Advanced Normalization Tools. The multi-stage registration pipeline consisted of rigid, affine, and symmetric normalization (SyN) transformations applied sequentially. For applying all transformations, linear interpolation was utilized during image resampling.

The primary image similarity metric across all registration stages was Mutual Information (MI), configured with 32 histogram bins and 25% regular sampling. We employed a multi-resolution approach with 5 levels. Registration optimization utilized a convergence criterion of 1e-6 with a convergence window of 10 iterations. The maximum number of iterations per level was set to 1000x500x250x100x0 for both the rigid and affine transformations, and 100x100x70x50x0 for the SyN transformation.

For the rigid and affine registration stages, a gradient step size of 0.1 was used. These stages applied smoothing with kernel sizes of 4x3x2x1x1 voxels and used shrink factors of 12x8x4x2x1. The subsequent SyN transformation employed a gradient step size of 0.1, an update field variance of 3 voxels, and a total field variance of 0 voxels (parameterized as SyN[0.1,3,0]). The SyN stage utilized smoothing kernels of 5x3x2x1x0 voxels and shrink factors of 10x6x4x2x1. Intensity winsorizing using the range [0.005, 0.995] was applied across all transformation stages to handle intensity outliers.

This hierarchical approach ensured accurate alignment while preserving critical anatomical features, with each stage building upon the previous transformation to achieve optimal registration quality.

2. Feature Extraction Parameters for Pyradiomics

In this study, MRI feature extraction was conducted using the open-source pyradiomics library with the following settings. For gray-level discretization, we employed a fixed bin width (binWidth) of 25. This method was selected over a fixed bin count to standardize the gray-level distribution across images, a crucial step for mitigating intensity variations that can arise from different MRI scanners or acquisition protocols. According to the Pyradiomics official documentation, this approach enhances the comparability and reproducibility of radiomics features. Gaussian smoothing was applied with sigma values of 3 and 5 to capture features at multiple scales. Images were normalized with a scaling factor of 100 to reduce intensity variations across different scans. For consistency in spatial resolution, images were resampled to an isotropic voxel spacing of [1, 1, 1] mm^3 using a B-spline (BSpline) interpolator. All available image types and features were enabled to comprehensively capture the relevant information.

3. Parameter Configuration and Training Strategy of the Clincal and Pyradiomics Model

To identify the most relevant and informative features, we implemented a multi-step feature selection strategy. Initially, the Mann-Whitney U test was applied to the training set to compare the distribution of each feature between different classes. The U test, a non-parametric method, is particularly well-suited for non-normally distributed data and is robust against outliers and small sample sizes. Subsequently, numerical features were standardized using z-scores, and categorical features were encoded using one-hot encoding. LASSO regression was then applied to further refine the feature selection process. LASSO incorporates an L1 regularization term into the loss function, which reduces the coefficients of many features to zero, thereby achieving sparsity and enhancing feature selection. The optimal regularization parameter λ was identified through five-fold cross-validation, which minimized the cross-validation error.

Considering the potential for class imbalance in the dataset to cause classification bias and inaccurate performance evaluation, we employed the SMOTE method to balance the data. SMOTE generates new minority class samples by interpolating in the feature space, thereby enhancing the representation of the minority class and producing a more balanced dataset. Five-fold cross-validation was then employed to identify the optimal parameters for the Support Vector Machine (SVM), including the regularization parameter C, kernel function types (e.g., linear, polynomial, radial basis function), and kernel coefficient gamma. Finally, predictions were generated by averaging the probabilities from an ensemble of five independently trained models.

4. Architectures of the Deep Learning Models

In the 2D model, the region of interest (ROI) was extracted from the largest tumor cross-section (n-th slice) in three MRI sequences: T1-weighted imaging (T1WI), T1-weighted contrast-enhanced imaging (T1CE), and T2-weighted imaging (T2WI). In the 2.5D model, two additional slices adjacent to the largest cross-section (n+2 and n-2 slices) were included, forming a three-slice image group to capture both local features and contextual information of the tumor. The image preprocessing steps involved resizing the images to 96×96 pixels, normalization, and tensor transformation to ensure consistency in the model input.

During the training phase, comprehensive data augmentation strategies were employed, including random flipping, rotation, and Gaussian noise addition, to enhance data diversity and mitigate the risk of overfitting. During the validation and testing phases, only basic preprocessing steps were applied. To address the issue of class imbalance, the ImbalancedDatasetSampler class from the PyTorch library was employed. This sampler assigns varying sampling weights to different classes, ensuring that each batch contains a balanced representation, thereby mitigating the impact of class imbalance on model training.

The three sequences were combined into a multi-channel input and subsequently passed through the backbone network. Two backbone networks were evaluated: the classical convolutional neural network model ResNet50 and the attention-based Swin Transformer. ResNet50 produced 2048 features per sequence, while Swin Transformer generated 768 features per sequence. The features from the three sequences were concatenated along the channel dimension, resulting in 6144 and 2304 features, respectively, for ResNet50 and Swin Transformer. These concatenated features were then fed into the

CBAM module, which applies channel and spatial attention mechanisms. Following this, the features were processed through a global average pooling layer and a fully connected layer, with the final output passed through a sigmoid function to obtain the probability of the sample being classified as positive.

5. Parameter Configuration and Training Strategy of the Deep Learning Models

All of the deep learning models were trained on an NVIDIA GeForce RTX 4090 GPU. For segmentation evaluation, we employed both Dice Similarity Coefficient (DSC) and Volumetric Similarity (VS) as complementary metrics. Volumetric Similarity (VS) is defined as[1]:

$$VS = 1 - \frac{|V_{\text{seg}} - V_{\text{GT}}|}{V_{\text{seg}} + V_{\text{GT}}}$$

While DSC measures spatial overlap between segmentations, VS specifically quantifies volumetric agreement, reflecting size accuracy. This dual-metric approach ensures comprehensive assessment of both positional and volumetric precision in automated meningioma segmentation.

The classification models were trained with a batch size of 32. Binary cross-entropy loss was employed as the loss function, while the Adam algorithm was utilized to optimize the model parameters. The initial learning rate was set to 0.0001. The ReduceLROnPlateau learning rate scheduler from PyTorch was utilized to dynamically adjust the learning rate. This scheduler monitors the minimum validation loss ('min') and reduces the learning rate by a factor of 0.1 if no decrease in validation loss is observed over 10 epochs. This performance-based adjustment strategy effectively prevents the model from becoming trapped in local minima and accelerates convergence toward a more optimal solution. Each change in the learning rate was logged to ensure transparency and control throughout the adjustment process.

Additionally, the training process utilized the validation set AUC as an early stopping criterion, terminating the training if no improvement in AUC was observed over 10 consecutive epochs. Five-fold cross-validation was adopted, with each fold generating an independent model. Final predictions were generated by averaging the probabilities across the ensemble of these five models.

6. Radiomics Quality Score (RQS) Assessment and CLAIM Guideline Compliance

We assessed our study using the Radiomics Quality Score (RQS) system proposed by Lambin et al. as a standardized tool for evaluating the quality of radiomics research. Our study achieved a score of 20 points.

The research demonstrated strength in several key areas: we provided comprehensive documentation of imaging protocols and parameters (2 points); employed multiple expert independent segmentation and annotation (1 point); implemented rigorous feature reduction methods to minimize overfitting (3 points); integrated non-radiomics features including demographic, imaging, and hematological parameters for multivariable analysis (1 point); discussed biological correlates, particularly the relationship between imaging features and Ki-67 expression (1 point); utilized the 5% threshold for Ki-67 analysis (1 point); provided thorough discrimination statistics including ROC curves, AUC values,

and confidence intervals (2 points); presented calibration curves in supplementary materials (1 point); performed validation on an independent external dataset from Centre 2 (4 points); compared our AI models with traditional clinical and radiomics models to demonstrate added value (2 points); and discussed the potential clinical utility in detail (2 points).

We acknowledge limitations in our study, primarily the absence of phantom studies, multiple time-point imaging analysis, prospective design, and cost-effectiveness analysis, which will be addressed in future research.

Checklist for Artificial Intelligence in Medical Imaging (CLAIM): 2024 Update

Section / Topic	No.	Item	Page / Line	No	NA
TITLE / ABSTRACT					
	**1**	Identification as a study of AI methodology, specifying the category of technology used (e.g., deep learning)	**1/1-2**		
ABSTRACT					
	**2**	Summary of study design, methods, results, and conclusions	**1/2-22**		
INTRODUCTION					
	**3**	Scientific and/or clinical background, including the intended use and role of the AI approach	**3/63-86**		
	**4**	Study aims, objectives, and hypotheses	**3/87-91**		
METHODS					
Study Design	**5**	Prospective or retrospective study	**4/94-101**		
	**6**	Study goal	**4/102-111**		
Data	**7**	Data sources	**4/91-98**		
	**8**	Inclusion and exclusion criteria	**4/94-101**		
	**9**	Data pre-processing	**4-5/115-134**		
	**10**	Selection of data subsets	**4/94-101**		
	**11**	De-identification methods	**4/112-113**		
	**12**	How missing data were handled	**5/140-141**		
	**13**	Image acquisition protocol	**4/115-116**		
Reference Standard	**14**	Definition of method(s) used to obtain reference standard	**4/102-111**		
	**15**	Rationale for choosing the reference standard	**4/102-111**		
	**16**	Source of reference standard annotations	**4/102-111**		
	**17**	Annotation of test set	**6/154-159**		

	**18**	Measures of inter- and intra-rater variability of features described by the annotators	**6/154-159**		
Data Partitions	**19**	How data were assigned to partitions	**8/205-210**		
	**20**	Level at which partitions are disjoint	**8/205-210**		
Testing Data	**21**	Intended sample size	**4/94-101**		

Section / Topic	No.	Item	Page / Line	No	NA
Model	**22**	Detailed description of model	**7/170-181**		
	**23**	Software libraries, frameworks, and packages	**7/195-201**		
	**24**	Initialization of model parameters	**7/170-176**		
Training	**25**	Details of training approach	**7/187-193**		
	**26**	Method of selecting the final model	**7/198-199**		
	**27**	Ensembling techniques			**NA**
Evaluation	**28**	Metrics of model performance	**8/198-199**		
	**29**	Statistical measures of significance and uncertainty	**8/200-201**		
	**30**	Robustness or sensitivity analysis	**9/245-256**		
	**31**	Methods for explainability or interpretability	**7/189-193**		
	**32**	Evaluation on internal data	**4/94-101**		
	**33**	Testing on external data	**4/94-101**		
	**34**	Clinical trial registration			**NA**
RESULTS					
Data	**35**	Numbers of patients or examinations included and excluded	**8/205-210**		
	**36**	Demographic and clinical characteristics of cases in each partition	**8/205-210**		
Model performance	**37**	Performance metrics and measures of statistical uncertainty	**9/219-256**		
	**38**	Estimates of diagnostic performance and their precision	**9/219-256**		
	**39**	Failure analysis of incorrect results		**No**	
DISCUSSION					
	**40**	Study limitations	**12/350-357**		

	**41**	Implications for practice, including intended use and/or clinical role	**12/341-349**		
OTHER INFORMATION					
	**42**	Provide a reference to the full study protocol or to additional technical details	**Suppleme ntary Methods**		
	**43**	Statement about the availability of software, trained model, and/or data	**Yes**		
	**44**	Sources of funding and other support; role of funders	**Yes**		

* Indicate page and/or line number for each checklist item that is present.   NA = not applicable.

Reference

1.Taha AA, Hanbury A(2015) Metrics for evaluating 3D medical image segmentation: analysis, selection, and tool. BMC Medical Imaging 15: 29.
